# Imidazo[1,2‑*a*]pyridines in Medicinal Chemistry: Recent Advances in Synthesis and Biological Activities

**DOI:** 10.1021/acsomega.5c08365

**Published:** 2026-01-06

**Authors:** Larissa A. P. Ferreira, Lucas Caruso, Nathalia F. Nadur, Daiana P. Franco, Gleyton L. S. Sousa, Renata B. Lacerda, Arthur E. Kümmerle

**Affiliations:** a Laboratory of Molecular Diversity and Medicinal Chemistry (LaDMol-QM), Department of Organic Chemistry, Institute of Chemistry, Federal Rural University of Rio de Janeiro, BR465, Km7, Seropédica,Rio de Janeiro 23897-000, Brazil; b Department of Pharmaceutical Sciences, Institute of Biological and Health Sciences, Federal Rural University of Rio de Janeiro, BR465, Km7, Seropédica,Rio de Janeiro 23897-000, Brazil

## Abstract

Imidazo­[1,2-*a*]­pyridines are widely recognized scaffolds present in several marketed drugs, including the anxiolytics alpidem, saripidem, necopidem, and zolpidem, which are some of the most prescribed medications for insomnia. In this review, we analyze publication trends, which reveal exponential growth in research involving this scaffold. We highlight recent synthetic strategies (2017–2025) for the preparation of imidazo­[1,2-*a*]­pyridine derivatives, such as condensation, multicomponent and tandem reactions, intramolecular cyclizations, and oxidative couplings under green conditions. In addition, we discuss innovative Medicinal Chemistry studies exploring their applications in the treatment of cancer, Alzheimer’s disease, tuberculosis, and neglected tropical diseases. Significant advances have been made in identifying derivatives with potent activity against specific biological targets, including kinases, tubulin, HDACs, the cytochrome bc1 complex of *Mycobacterium tuberculosis*, and key enzymes involved in the pathogenesis of Alzheimer’s disease, such as cholinesterases and secretases. Altogether, this review consolidates the vast therapeutic potential of the imidazo­[1,2-*a*]­pyridine core, emphasizing its synthetic versatility and broad spectrum of biological activities, which firmly establish it as a privileged scaffold for drug discovery.

## Graphical abstract

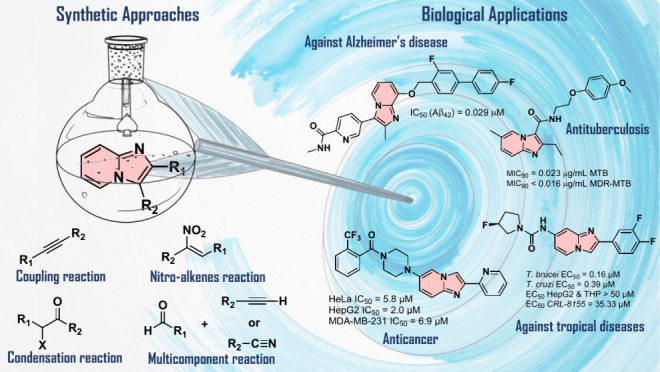

## 1 Introduction

Heterocycles are highly promising compounds found in several bioactive natural products, including pharmaceuticals, agrochemicals, dyes, and many others.^1^ Among these compounds are the imidazopyridines (IMs), defined as nitrogenous heterocycles that have an imidazole ring fused to a pyridine ring.^2^ The combination of its nitrogen-containing heterocycle and synthetic facility makes IMs a widespread explored nucleus presenting many pharmacological actions, many times used as central nucleus and others only as linkers.^3^ Among the activities, the main relates are antibacterial, anticancer, antiviral, anti-inflammatory, antitumor, antiparasitic, antipyretic, antitubercular, and analgesics, in the treatment of hepatitis C and HIV.^4–7^ Recently, interesting studies related to the inhibition of β-amyloid formation and GABA receptor agonists have been shown.^3^ We can also find IM applications in the chemistry of transition metals, due to its high tendency of ortho donation in *N*-heterocycle carbenes, as well as applications in fluorescence, because of their intramolecular proton transfer properties in the excited state, acting as sensors, laser dyes, and molecular switches.^8^

This class of molecules is known for its different isomers: imidazo­[1,2-*a*]­pyridines (**1**), imidazo­[1,5-*a*]­pyridines (**2**), imidazo­[4,5-*b*]­pyridines (**3**), and imidazo­[4,5-*c*]­pyridines (**4**), classified according to the position of the nitrogen atoms resulting in different imidazole and pyridine rings and each one with its characteristics and physicochemical properties (Figure 1).^3^

**Figure 1:**
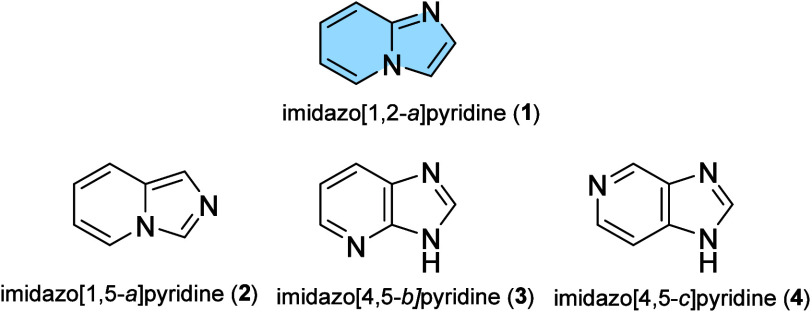
Imidazopyridine isomers.

The focus of this review, however, is the imidazo­[1,2-*a*]­pyridine isomer (**1**) that accounts for the largest number of commercially available drugs, discussed below in the text, and has been the subject of extensive research in medicinal chemistry, highlighting its broad spectrum of reported biological activities.^9^ To provide an overview of the global research landscape on imidazo­[1,2-*a*]­pyridines, we conducted a search in the Scopus database using the term “imidazo­[1,2-*a*]­pyridine” in the article titles, abstracts, or keywords. From 1954 to the end of 2024 (the last fully completed year), approximately 2670 articles on this azaheterocycle were published (Figure 2A). The publication trend follows an apparent exponential growth curve with the number of studies remaining relatively flat for decades before increasing sharply after 2005.

**Figure 2:**
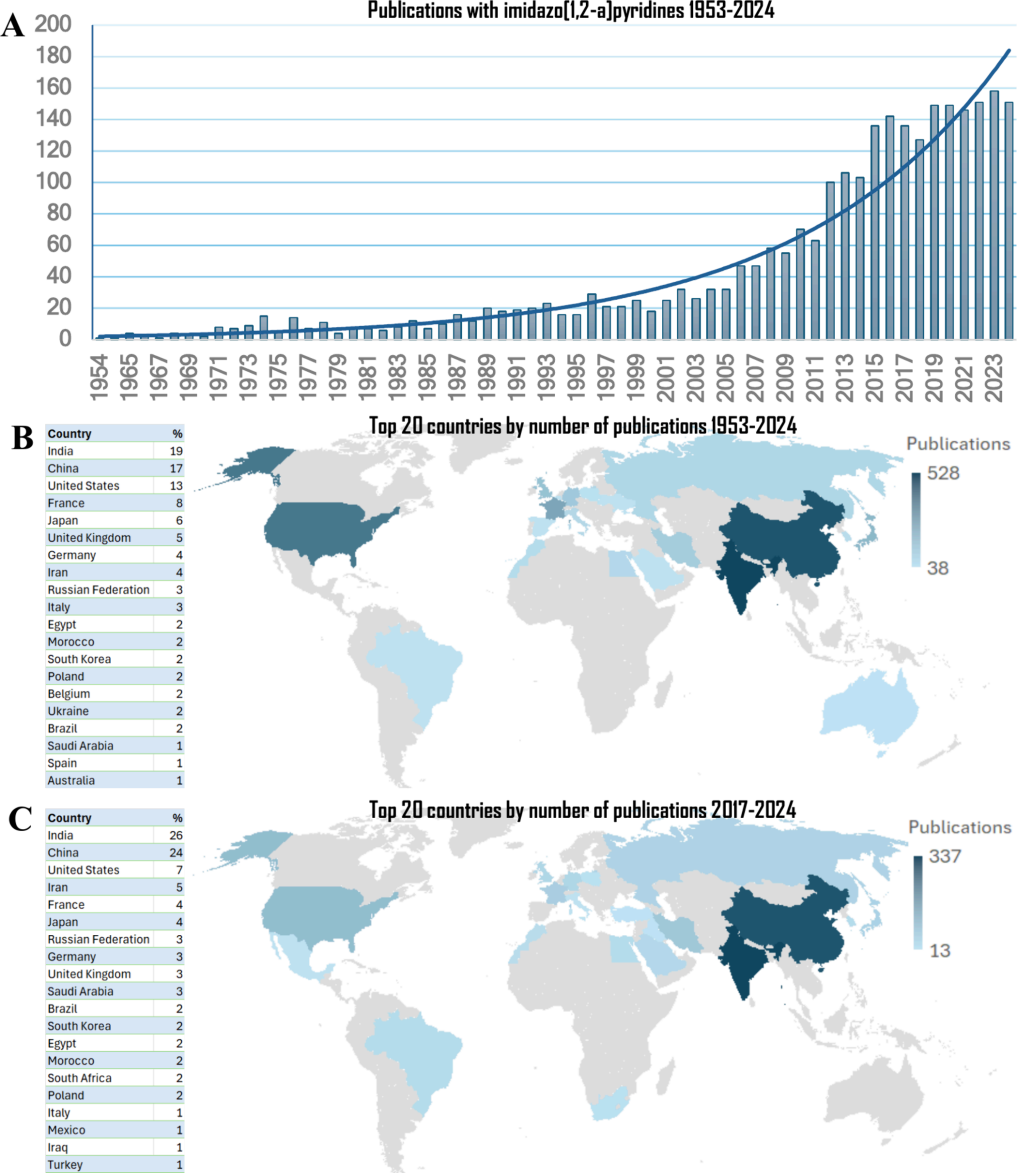
Citations and publications over time. Result obtained from a search in the Scopus database on August 1, 2025, for publications containing the term “imidazo­[1,2-*a*]­pyridines” within article title, abstract, or keywords. (A) Number of publications by year from 1954 to 2024; (B) list of top 20 countries by number of publications and representative percentual from 1954 to 2024; (C) list of top 20 countries by number of publications and representative percentual from 2027 to 2024.

India, China, and the United States were the leading countries over the entire period, accounting for 19, 17, and 13% of total publications, respectively. During the time frame of this review (2017–2024), the ranking of these countries remained unchanged; however, the share of publications from India and China increased to 26 and 24% while that of the United States declined to 7%, about half of its overall contribution in the full period. Another notable change was the decrease in France’s share from 8 to 4%, shifting its position from fourth to fifth (Figure 2B,C).

An analysis of the subject areas of publications on imidazo­[1,2-*a*]­pyridines shows that the 2017–2024 (the last fully completed year) period did not significantly alter the main research areas or the overall representativeness of this scaffold (Figure 3). Chemistry remains the leading field, accounting for 38–40% of publications depending on the period, followed by Biochemistry (18–19%) and Pharmacology (15–19%) (Figure 3). The high proportion of studies in these biological areas underscores the importance of imidazo­[1,2-*a*]­pyridines in studies involving bioactivity and Medicinal Chemistry research, which are the central focus of this review alongside reported synthetic methods for this heterocycle.

**Figure 3:**
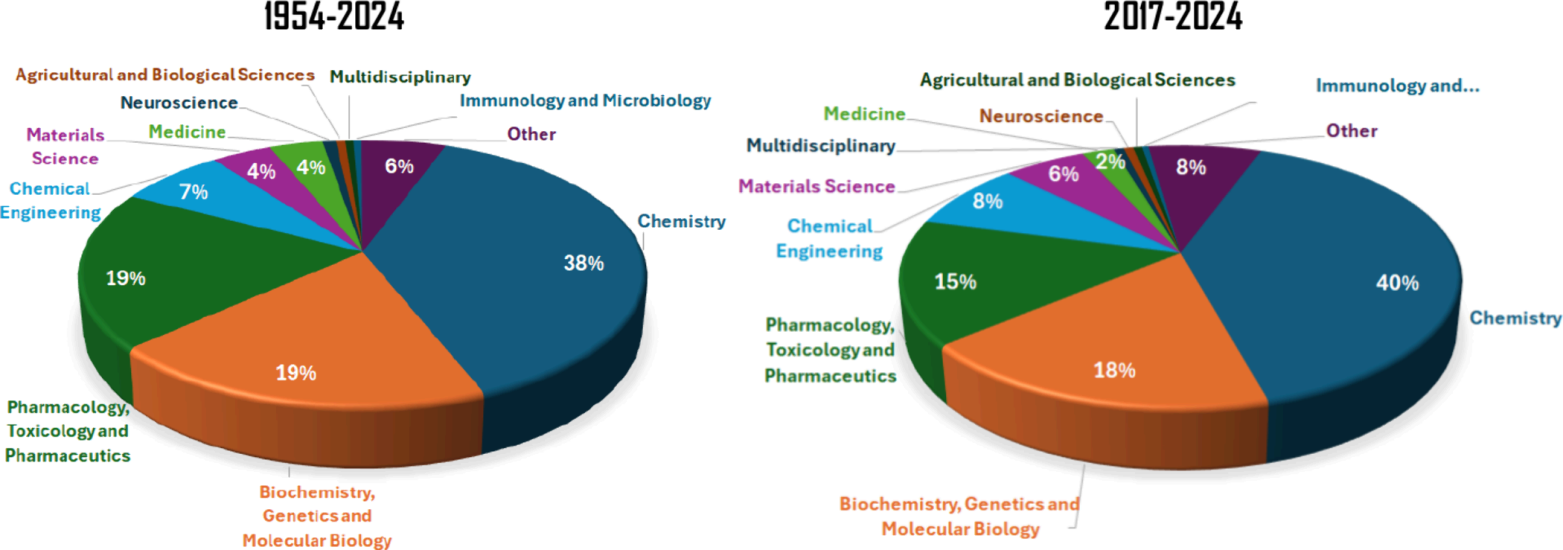
Percentual of documents by subjected area by period obtained from a search in the Scopus database on August 1, 2025, for publications containing the term “imidazo­[1,2-*a*]­pyridines” within article title, abstract, or keywords.

As mentioned above, imidazo­[1,2-*a*]­pyridine is a widely explored scaffold, and due to its broad pharmacological applications, this structure is also present in various anxiolytic drugs, e.g., alpidem (**5**), saripidem (**6**), necopidem (**7**), and zolpidem (**8**), one of the most popular medications for treating insomnia. Indeed, due to acts in various central nervous system disorders (CNS), IMs are also called by “non-benzodiazepines” because despite being structurally different from benzodiazepines, their pharmacological properties are quite similar.^10,11^ Additionally, non-CNS imidazo­[1,2-*a*]­pyridine drugs can also be cited as olprinone (**9**) (cardiotonic agent), zolimidine (**10**) (gastroprotective), miroprofen (**11**) (analgesic and nonsteroidal anti-inflammatory), and minodronic acid (**12**) (osteoporosis) (Figure 4).^6,8,12,13^

**Figure 4:**
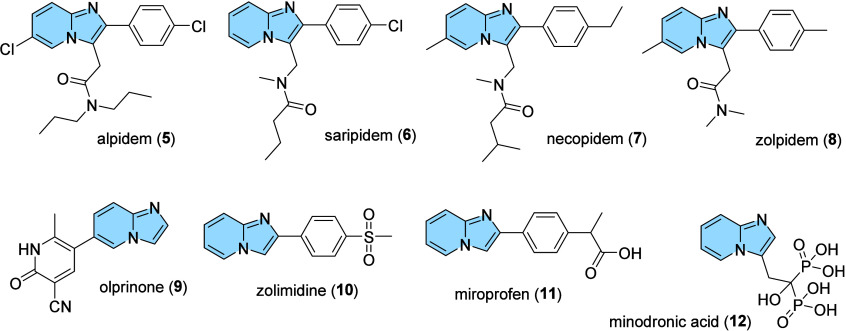
Structure of available drugs containing an imidazo­[1,2-*a*]­pyridine nucleus.

While recent studies have addressed the synthesis and functionalization of imidazopyridines, the literature still lacks comprehensive reviews that thoroughly explore the pharmacological applications of this scaffold.^14–20^ Due to its importance in medicinal chemistry, there are several synthetic strategies to produce imidazo­[1,2-*a*]­pyridine derivatives, such as through condensation; multicomponent; and tandem reactions, intramolecular cyclization, and oxidative couplings.^1^ In this review, we will address some of the most recent examples (2017–2025) for the synthesis of imidazo­[1,2-*a*]­pyridines, also presenting and discussing some current innovative Medicinal Chemistry studies for the treatment of cancer, Alzheimer’s disease, tuberculosis, and parasitic diseases using this scaffold.

## 2 Methods of Obtaining Imidazo[1,2-*a*]pyridines

Many studies related to imidazo­[1,2-*a*]­pyridines synthesis have been published in recent years.^21^ We conducted a search in the Scopus database using the term imidazo­[1,2-*a*]­pyridine in article titles, abstracts, or keywords, with subject area filter “Biochemistry, Genetics and Molecular Biology”, “Pharmacology, Toxicology and Pharmaceuticals”, “Chemistry” or “Multidisciplinary”, and document type filter “Article”. Several approaches with reactional modifications/adaptations have been used in the past decade, and these methodologies could be classified into six main groups: condensation reaction, multicomponent reactions, reaction with nitro-alkenes, cross-coupling reaction, intramolecular cyclization reaction, under green conditions, and others (Figure 5).^10,22^

**Figure 5:**
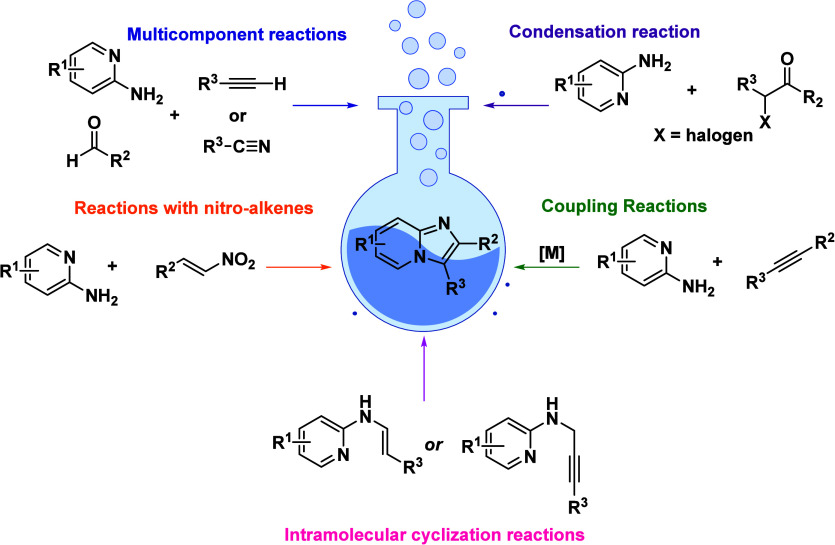
Overview of synthetic methodologies for obtaining imidazo­[1,2-*a*]­pyridines.

### 2.1 Condensation Reaction

The classical condensation reaction of α-halocarbonyl derivatives with 2-aminopyridine furnishing imidazo­[1,2-*a*]­pyridines was first described by Tschitschibabin (also known as Chichibabin) in 1925.^23^ Chichibabin’s research focused on the synthesis of substituted pyridines through the condensation of carbonyl compounds with ammonia. One of the reactions involved the combination of 2-aminopyridine with bromoacetaldehyde at 150–200 °C (yield 20%), leading to the formation of imidazo­[1,2-*a*]­pyridines (Figure 6A), and its proposed mechanism reaction^24,25^ is described in Figure 6B. Because of Chichibabin’s seminal studies, several adaptations were made to improve the efficiency of the reaction.^10,23,26^

**Figure 6:**
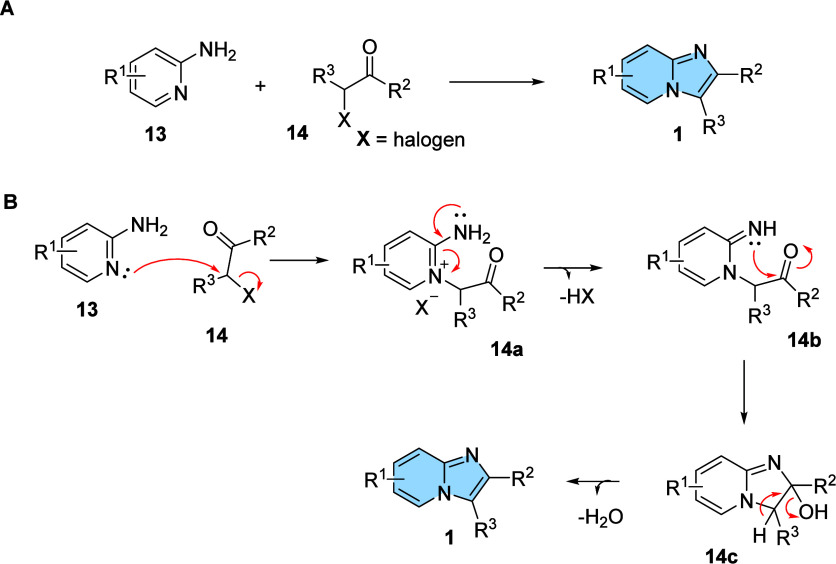
(A) General scheme for the synthesis of imidazo­[1,2-*a*]­pyridines from condensation reactions. (B) Proposal of mechanism reaction for the formation of imidazo­[1,2-*a*]­pyridines from condensation reactions.^24,25^

Among adaptations made for the synthesis of imidazo­[1,2-*a*]­pyridines, many of them explored the same intramolecular condensation reaction of pyridine precursors (such as 2-aminopyridine) with different α-haloketones.^2^ However, many other condensation reactions have employed different electrophilic reactants in place of α-haloketones. The main examples are α-diazo,^27^ 1,3-dicarbonyl,^28^ styrene,^29^ haloalkynes,^30^ and arylglyoxal hydrate compounds,^31^ among others that can be explored for the synthesis of these heterocycles.

The obtention and isolation of α-halocarbonyl compounds sometimes is struggling. Taking this problem into account, Bhagat and Telvekar developed the synthesis of imidazo­[1,2-*a*]­pyridine-3-carboxylate compounds from an elegant, simple, and ecological one-pot reaction using 1-bromo-2,5-pyrrolidinedione (NBS). The procedure explored the production of an *in situ* α-brominated intermediate from ketones, diketones, and β-keto-esters, followed by reaction with the nucleophile (2-aminopyridine, **13**), resulting in the bicyclic heterocycles fused with imidazole. Many reaction condition evaluations, including different proportions of chemicals and solvent, temperature, and time modifications, were made. From this optimization, the protocol using 1.2 equiv of NBS at 80 °C in water was ideal for transformations (Figure 7A).^28^

**Figure 7:**
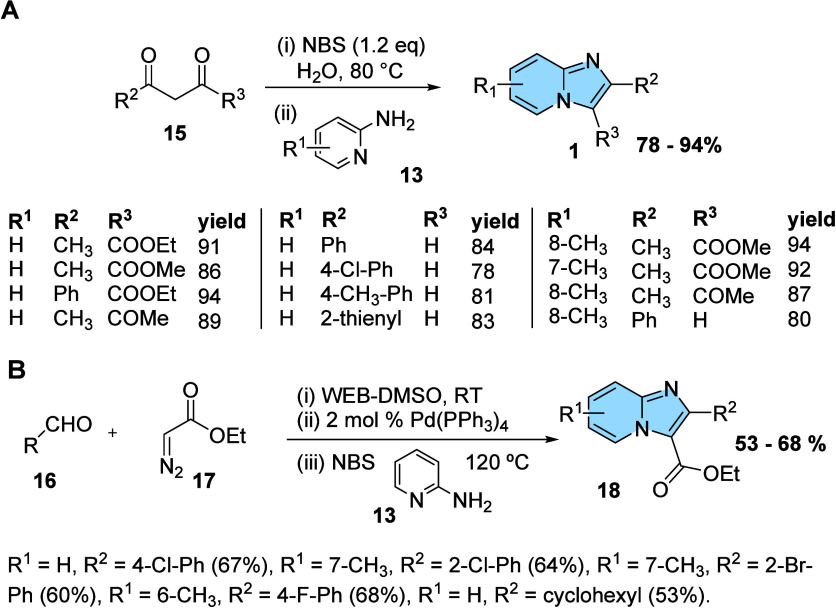
One-pot synthesis of imidazo­[1,2-*a*]­pyridine-3-carboxylates (A) from several dicarbonyl compounds and aminopyridines and (B) from α-diazo-β-hydroxyester conversion to β-keto-esters.

As the first example, a one-pot reaction was applied by Dutta et al. in the synthesis of imidazo­[1,2-*a*]­pyridine-3-carboxylates. Initially, they developed an aldol condensation system for the synthesis of α-diazo-β-hydroxyesters using a mixture of water extract of banana ash and dimethyl sulfoxide (WEB-DMSO) (i) as a solvent, based on studies with many solvents.

The WEB has basic character and has been used as an alternative reaction method or catalytic solvent system for various organic transformations. It can be used alone or, in some cases, mixed with organic solvents to increase the miscibility of reagents to provide higher yields. Optimization studies carried out identified WEB-DMSO (1:1), room temperature, and 2 mol % Pd­(PPh_3_)_4_ as the best conditions. The protocol explored the 1,2-hydrogen migration catalyzed by palladium for the conversion of α-diazo-β-hydroxy esters (formed in **i**) to β-keto-esters (**ii**). Then, β-keto-esters were converted to imidazo­[1,2-*a*]­pyridine-3-carboxylates **18** using *N*-bromosuccinimide (NBS) and 2-aminopyridines (**iii**) (Figure 7B), obtaining compounds with good yields.^27^

The next one-pot synthesis proposed a regioselective one-pot synthesis of 3-fluoro-imidazo­[1,2-*a*]­pyridines (F-IMPY) from styrene and 1-fluoropyridinium tetrafluoroborate (NPy-BF) as both a fluorine source and a base. Different from two first examples, NBS here plays a dual role: oxidant and source of bromine. Four transformations take place from styrene in three steps to provide F-IMPY: bromination and oxidation (**i**), condensation (**ii**), and fluorination (**iii**) (Figure 8). The reaction was optimized by Said and his research group, who identified *tert*-BuOH and water (2:1) as the optimal solvent mixture. The reactivity of various fluorinating agents was then investigated, with NPy-BF identified as being the most effective. Finally, the reaction scope was evaluated, demonstrating broad substrate applicability and functional group tolerance for styrenes **19** and 2-aminopyridines **13**, giving the 3-fluoro-imidazo­[1,2-*a*]­pyridines **20** in good yields of up to 82%.^29^

**Figure 8:**
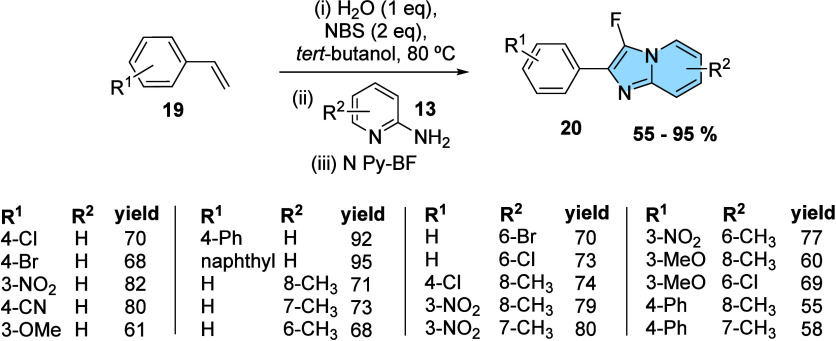
One-pot synthesis of 3-fluoro-imidazo­[1,2-*a*]­pyridines from styrenes.

### 2.2 Multicomponent Reactions

Multicomponent reactions (MCRs) offer great process simplicity and economy by combining three or more readily available substrates to generate complex molecules. They present several advantages over conventional transformations, including the formation of multiple bonds in just a few steps and the rapid production of target compounds without the need to isolate intermediates.^10,32,33^

There are several methodologies to synthesize imidazo­[1,2-*a*]­pyridines through MCRs. One of the most important and best known is the multicomponent cycloaddition [4 + 1] Groebke-Blackburn-Bienayme reaction (GBB). The synthesis is based on the condensation of aldehydes, amidines, and isocyanides in the catalysis of a Lewis or Bronsted acid (Figure 9).^34–37^ There are also copper-catalyzed 4-component isocyanide reactions;^38^ reactions of aminopyridines, aldehydes with alkynes,^39–41^ and alkyne carboxylic acids;^40^ and reaction with nitroalkanes and among other derivatizations.^2^

**Figure 9:**
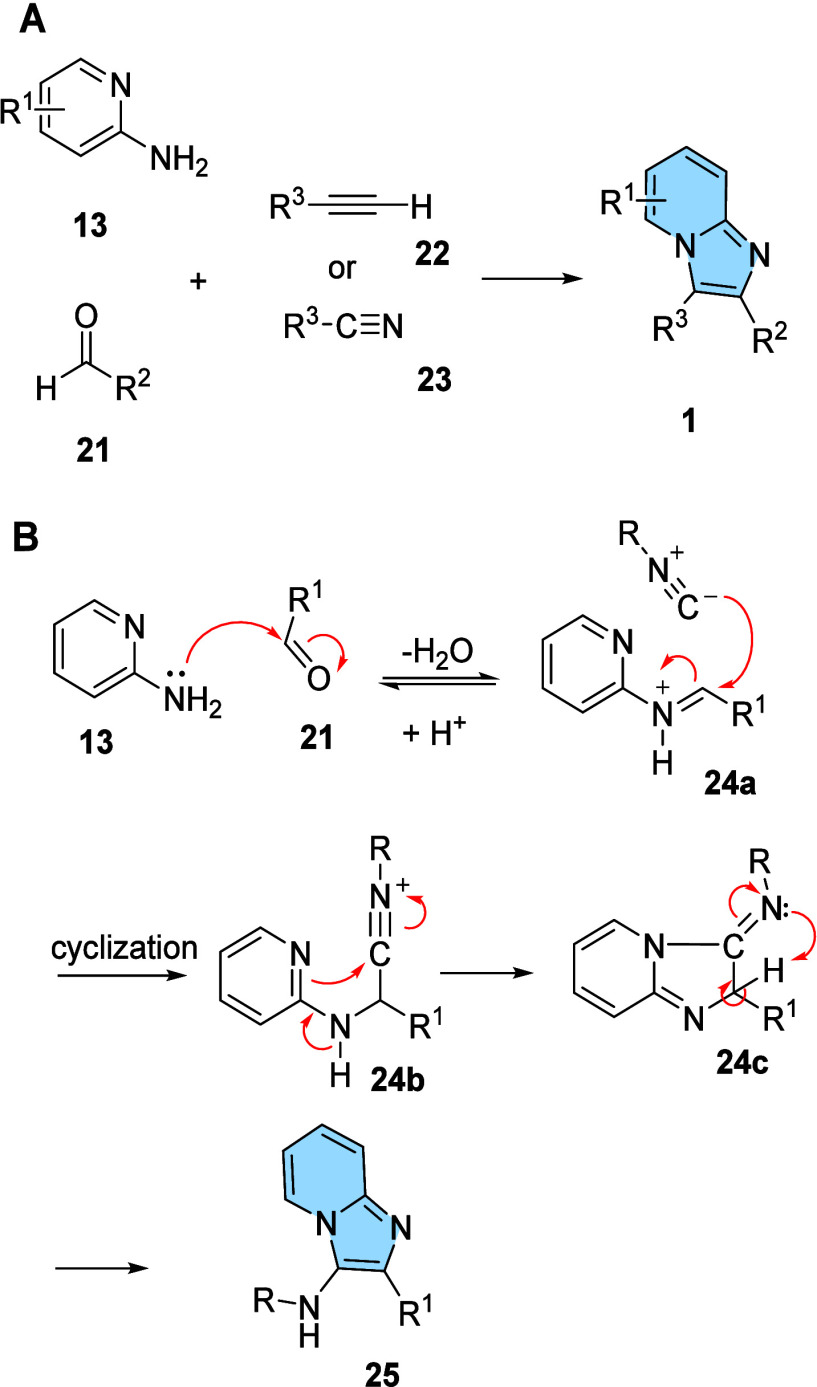
(A) General synthesis of imidazo­[1,2-*a*]­pyridines from multicomponent reactions. (B) Proposal of a mechanism reaction for the formation of 3-amino-imidazo­[1,2-*a*]­pyridine derivatives **25** through the GBB multicomponent reaction.

The proposed general mechanism for the formation of the imidazo­[1,2-*a*]­pyridines (**25**) through the multicomponent GBB reaction involves the formation of the iminium ion (**24a**) followed by a nucleophilic attack for the isonitrile generating **24b** (Figure 9). The electron pair of pyridine nitrogen is responsible for the intramolecular attack generating the heterocyclic intermediate **24c**, whose rearomatization provides the desired imidazo­[1,2-*a*]­pyridine derivatives **25.** ^42^

The methodology described by Groebke-Blackburn-Bienayme was explored in 2014 by Lacerda and collaborators as a key step to obtain the imidazo­[1,2-*a*]­pyridine nucleus in the synthesis of new *N*-glycinylhydrazone derivatives (**30**) as inhibitors of the production of TNF-α. The MCR between 2-aminopyridine (**13**), benzaldehyde (**21**), or pivalaldehyde (**26**) and ethyl isocyanoacetate (**27**) in ethanol at room temperature was used to obtain the ester intermediate (**28**), which was further derivatized to obtain the bioactive compounds imidazo­[1,2-*a*]­pyridines-*N*-glycinylhydrazones (**30**), demonstrating the importance and versatility of Groebke’s MCR for Medicinal Chemistry (Figure 10).^43^

**Figure 10:**
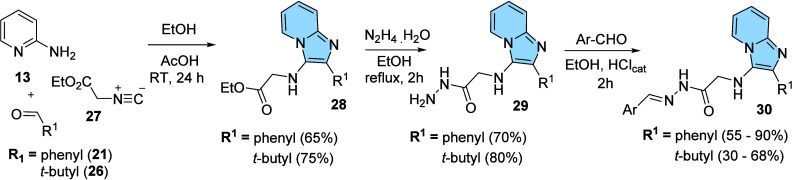
Synthesis of anti-TNF-α *N*-glycinylhydrazones-imidazo­[1,2-*a*]­pyridines from a multicomponent reaction with isonitriles.

Although classical MCR conditions typically employ ethanol as the solvent and AcOH as the catalyst, the following four examples describe adapted protocols that use alternative solvents and catalytic systems. In the first one, Zadmard developed a method for the synthesis of 2,3-substituted imidazo­[1,2-*a*]­pyridines **32** via GBB reaction, using calix[6]­arene-SO_3_H as the catalyst and water as solvent. Foremost, it was evaluated the reaction of 2-aminopyridine **13**, benzaldehyde **21**, and cyclohexyl isocyanide **31** with different calix­[n]­arenes catalysts, solvents, and the ideal temperature. The calix[6]­arene-SO_3_H and water were chosen as catalyst and solvent at 25 °C. The use of calix­[n]­arenes in water provided a hydrophobic cavity aiding in the solubility of the reaction, since the disadvantage of using water as a solvent is the hydrophobicity of organic and water-insoluble compounds. This catalyst system is recoverable with a simple extraction using an organic solvent and reusable for at least 5 cycles without loss of activity. After the reaction optimization, different derivatives with modifications in 2-aminopyridine, benzaldehyde, and isocyanides were evaluated (Figure 11A), showing good to excellent yields and relatively short reaction time.^35^

**Figure 11:**
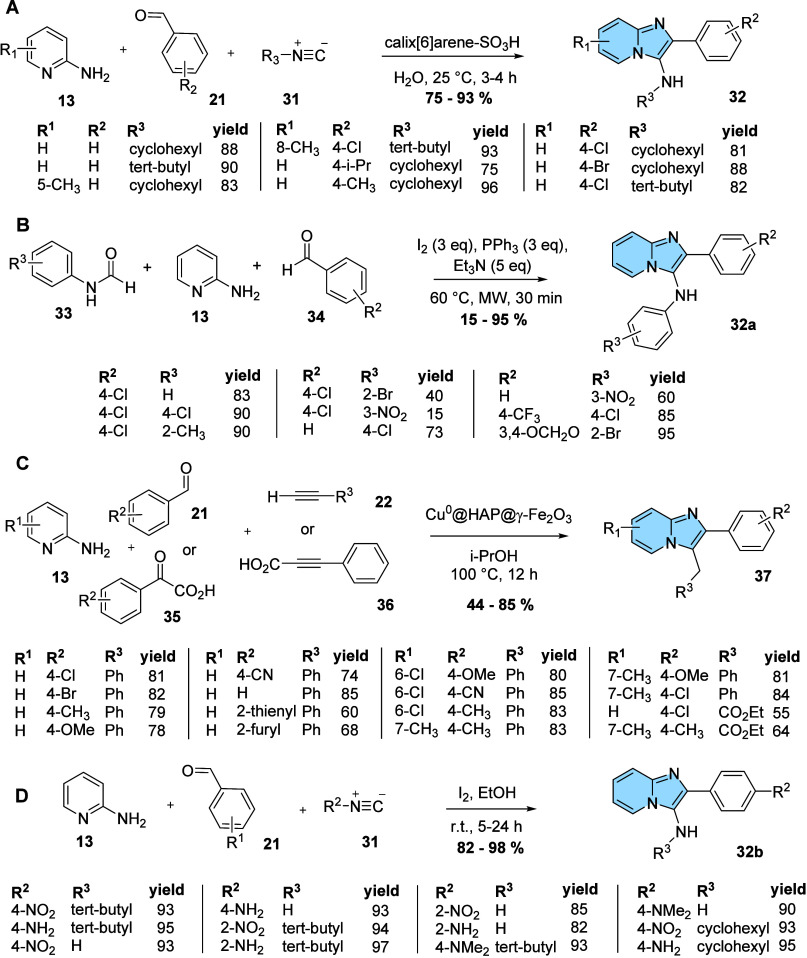
Adapted multicomponent GBB reactions. (A) Synthesis of imidazo­[1,2-*a*]­pyridines (**32**) using Bronsted acid calix[6]­arene-SO_3_H in water as a catalyst. (B) One-pot synthesis of compound imidazo­[1,2-*a*]­pyridine (**32a**) catalyzed by I_2_-PPh_3_-Et_3_N. (C) Synthesis of imidazo­[1,2-*a*]­pyridines (**37**) catalyzed by copper with doped magnetic hydroxyapatite nanoparticles (Cu^0^@HAP@γ-Fe_2_O_3_). (D) Synthesis of imidazo­[1,2-*a*]­pyridines through iodine-catalyzed three-component condensation.

In the second, Kaur delineates a one-pot methodology using a reagent system with iodine, triphenylphosphine, and triethylamine (I_2_-PPh_3_-Et_3_N) to convert *N*-formylamine (**33**), 2-amino pyridine (**13**), and aldehyde (**34**) into 2,3-substituted imidazo­[1,2-*a*]­pyridine (**32a**) and undergo a GBB multicomponent reaction. This methodology occurs in two steps; at first, the phenyl isocyanide is formed through the conversion of *N*-formylamine with I_2_-PPh_3_-Et_3_N in dichloromethane at room temperature in 2 h. This process leads to the formation of hydroiodic acid (HI) in the reaction that could be used simultaneously to catalyze the GBB reaction. The second step is the addition of 2-aminopyridine and aldehyde to the same reaction mixture, using microwave irradiation at 60 °C for 30 min. The condition reaction was initially tested for compound **32a** (Figure 11B), and then the other 14 compounds were synthesized with good yields from 15 to 95%.^36^

The third example describes the one-pot multicomponent synthesis of imidazo­[1,2-*a*]­pyridines (**37**) from 2-aminopyridine derivatives (**13**), aldehydes (**21**), or glyoxylic acids (**35**) and alkynes (**22**) or alkyne carboxylic acid (**36**) catalyzed by Cu(0) incorporated magnetic hydroxyapatite nanoparticles (Cu^0^@HAP@γ-Fe_2_O_3_). Primarily, the preparation and testing of the catalysts were carried out, involving several processes (ion exchange, hydrolysis, and oxidation). First, the hydroxyapatite (Ca_10_(PO_4_)_6_(OH)_2_) is encapsulated with tri-iron tetroxide, which is calcined to form the desired compound HAP@ϒ-Fe_2_O_3_. Then, the incorporation of copper occurs through the reduction of a solution of Cu^2+^. This process was characterized by several methods such as Fourier transform infrared spectroscopy, scanning electron microscopy, and energy scattering X-ray spectroscopy, among others. The second step was the identification of the reaction conditions, finding the best results using isopropyl alcohol (i-PrOH) as a solvent, at 100 °C. The multicomponent reaction of 2-aminopyridine was carried out with a variety of aryl aldehydes and phenylacetylenes (Figure 11C) to evaluate the effectiveness of the catalyst. Transition metal-based nanocatalysts are promising and efficient; in this case, the catalyst not only increases the reaction activity of 2-aminopyridine, aryl aldehydes, and alkynes but also leads to the decarboxylative coupling of alkyne carboxylic acid and/or glyoxylic acid to the synthesis of imidazo­[1,2-*a*]­pyridines (**37**) obtaining good yields, and it can be easily separated from the process and reused.^40^

In the fourth example (Figure 11D), a simple and efficient iodine-catalyzed method for synthesizing imidazo­[1,2-*a*]­pyrazines and imidazo­[1,2-*a*]­pyridines with fluorescent and anticancer properties was proposed by Krishnamoorthy. Products were obtained in excellent yields through an isocyanide-based Ugi-type three-component reaction using *tert*-butyl isocyanide (**31**), aryl aldehyde (**21**), 2-aminopyridine (**13**) or 2-aminopyrazine, and iodine catalyst in ethanol at room temperature.^44^

### 2.3 Reactions with Nitro-alkenes

Nitro-alkenes (**38**) are very useful reactants for the obtention of imidazo­[1,2-*a*]­pyridines (**40**) through the reaction with 2-amino-pyridine (**13**), giving an interesting molecular diversification depending on the chosen reactants and protocols.^45–48^ The first use of nitro-alkenes for the synthesis of imidazo­[1,2-*a*]­pyridines was described by Nair and colleagues in 2012, so recently. They showed that Morita-Baylis-Hillman (MBH) acetates of nitro-alkenes (**38**) react with different aminopyridines (**13**) in methanol originating 2-aryl-imidazo­[1,2-*a*]­pyridines (**40**). Moreover, this protocol presented excellent yields, regioselectivity, and usefulness to obtaining the so-called imidazo­[1,2-*a*]­pyridine drugs alpidem (**5**) and zolpidem (**8**) (Figure 12A). The proposed mechanism starts throwing a Michael addition from the primary amine of 2-aminopyridine to the electrophilic moiety of MBH (**39**), leading to an elimination of acetate in a S_N_2′ mechanism pathway. In the second step, intermediate **40a** is implicated in a regioselective 5-exo-trig cyclization, originating **40b**, which after elimination of HNO_2_ affords the final 2-aryl-imidazo­[1,2-*a*]­pyridine (**40**) (Figure 12B).

**Figure 12:**
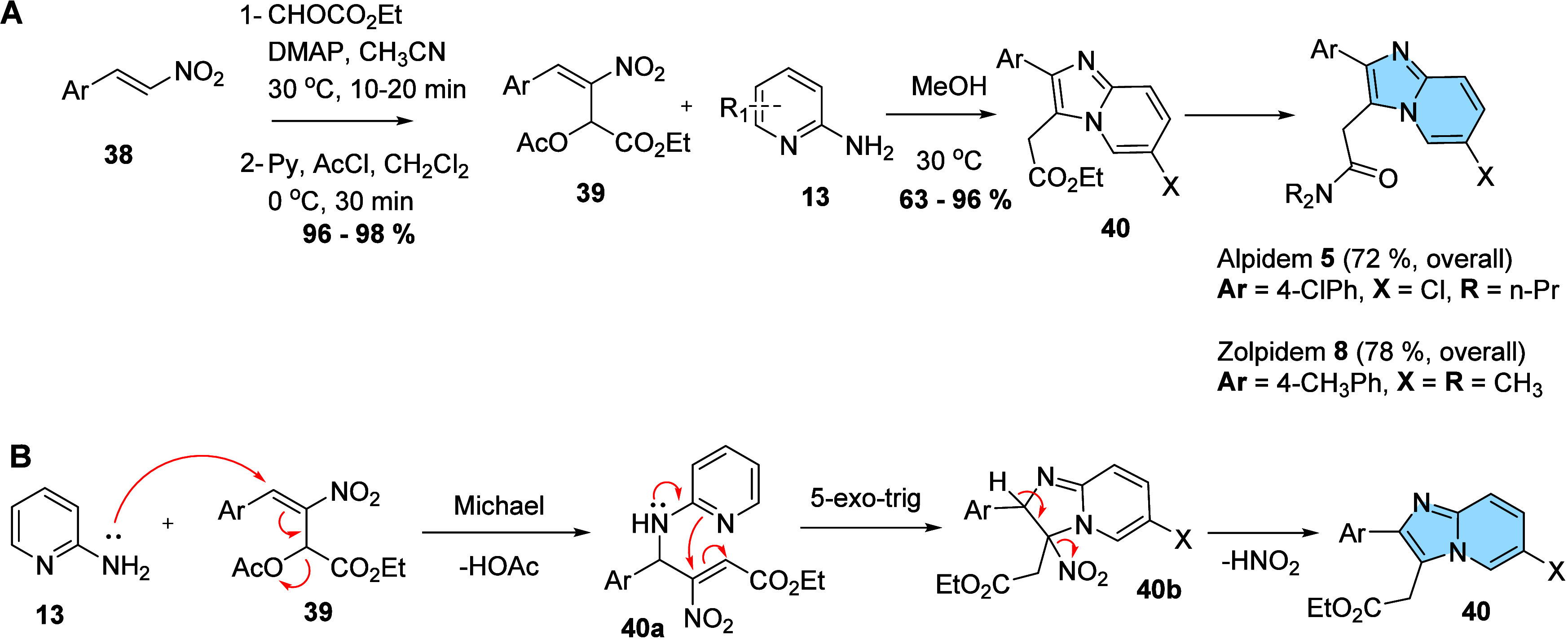
(A) General scheme of synthesis of first imidazo­[1,2-*a*]­pyridines (**40**) from 2-amino-pyridine (**13**) and nitro-alkenes (**38**). (B) Proposed mechanism for the formation of 2-aryl-imidazo­[1,2-*a*]­pyridines (**40**).^48^

Another protocol for the synthesis of 2-aryl-imidazo­[1,2-*a*]­pyridines was described by Gupta and his research group. They synthesized novel 2,5,7-triaryl-imidazo­[1,2-*a*]­pyridine-8-carbonitrile derivatives (**42**) from 2-amino-4,6-diaryl-pyridine-3-carbonitrile (**41**) and β-nitro-styrenes (**38**) using FeCl_3_ as a Lewis acid catalyst in a solvent-free methodology, obtaining excellent yields (71–76%). The authors evaluated the influence of different solvents (DMF, CHCl_3_, EtOH, etc.), catalysts, reaction times, and temperature. Finally, they found that the methodology without solvent with 20 mol % catalyst (FeCl_3_) provided the best results (Figure 13). Thirteen new derivatives were synthesized and their anticancer activities evaluated against lung, pancreas, breast, and colon cancer cells.^49^

**Figure 13:**
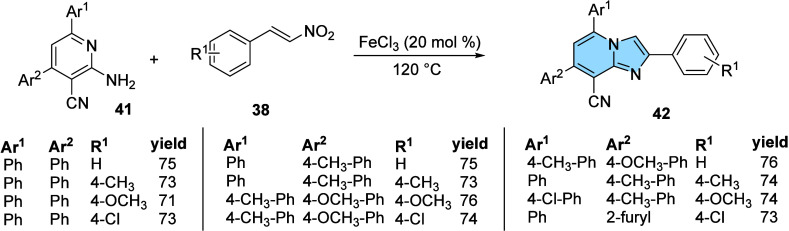
Synthesis of 2,5,7-triaryl-imidazo­[1,2-*a*]­pyridine-8-carbonitrile derivatives (**42**).

Nitro-alkenes (**38**) are also applied to the synthesis of regioisomeric 3-aryl-imidazo­[1,2-*a*]­pyridine derivatives. Devi and co-workers developed a regioselective protocol for obtaining several 3-aryl-imidazo­[1,2-*a*]­pyridines (**43**) from β-nitro-styrenes (**38**) and substituted aminopyridines (**13**) using a base and oxidant at room temperature (Figure 14A). First, solvents with different polarities (CH_3_CN, DMF, H_2_O, toluene, CH_2_Cl_2_, etc.), organic and inorganic bases (Et_3_N, piperidine, K_2_CO_3_, *t*-BuOK, etc.), and different peroxides (H_2_O_2_, TBHP, etc.) were investigated. The most effective methodology found was Et_3_N (10 mol %) and H_2_O_2_ (3 equiv) in CH_2_Cl_2_ at 25 °C. Subsequently, the versatility of the methodology was evaluated showing that a variety of β-nitro-styrenes (**38**) and substituted aminopyridines (**13**) could be combined, forming several 3-aryl-imidazo­[1,2-*a*]­pyridines (**43**) with moderate to high yields (54–83%).^50^ The reason for this reaction furnishes the 3-aryl-imidazo­[1,2-*a*]­pyridine regioisomer, despite starting with reactants similar to the two first examples, is explained by the basic/oxidant media, and is depicted in the proposed mechanism in Figure 14B. Initially, the presence of a base in the media increases the nucleophilicity and favors the intermolecular Michael addition from the pyridine nitrogen. The intermediate **44a** undergoes intramolecular cyclization to generate intermediate **44b** that suffers an oxidative elimination process, generating 3-aryl-imidazo­[1,2-*a*]­pyridine regioisomer **43** (Figure 14B).

**Figure 14:**
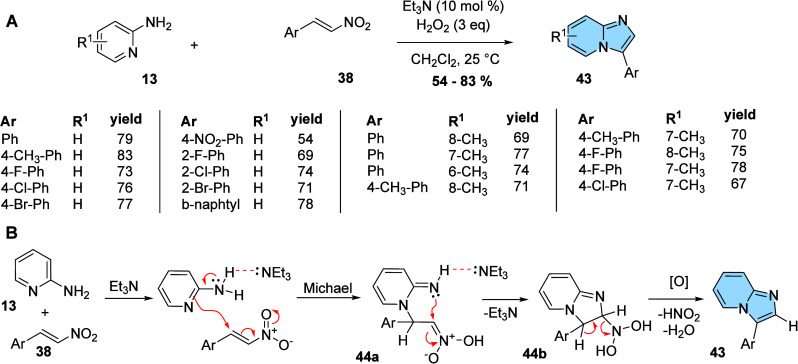
(A) Synthesis of 3-aryl-imidazo­[1,2-*a*]­pyridines (**43**) from several β-nitro-styrenes (**38**) and aminopyridines (**13**). (B) Proposed mechanism for the formation of 3-aryl-imidazo­[1,2-*a*]­pyridines (**43**).^48,50^

Also, in 2018, another example of 3-aryl substituted imidazo­[1,2-*a*]­pyridine regioisomers obtention was described. Tzani and his research group used a heterogeneous catalyst of gold nanoparticles supported on alumina (Au/Al_2_O_3_) in the regioselective synthesis of 2-nitro-3-aryl-imidazo­[1,2-*a*]­pyridines (**45a**) (yields ranging from 74 to 91%), followed by chemoselective reduction, forming several 2-amino-3-aryl-imidazo­[1,2-*a*]­pyridines (**45b**) in high yields (Figure 15). The authors showed that the catalyst is effective for both the synthesis of the imidazo­[1,2-*a*]­pyridine nucleus and the reduction of the nitro group, being also possible to directly obtain the 2-amino-3-aryl-imidazo­[1,2-*a*]­pyridine (**45b**) in a two-step one-pot reaction. Moreover, they found that the catalyst can be recycled twice in the synthesis of 2-nitro-3-aryl-imidazo­[1,2-*a*]­pyridine (**45a**) and up to five times in the reduction step without yield loss.^51^

**Figure 15:**
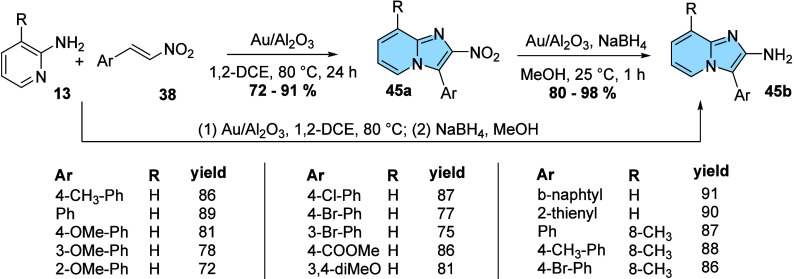
Synthesis of 2-nitro-3-aryl-imidazo­[1,2-*a*]­pyridines (**45a**) and 2-amino-3-aryl-imidazo­[1,2-*a*]­pyridines (**45b**) catalyzed by Au/Al_2_O_3_.

### 2.4 Coupling Reactions

The use of oxidative cross-coupling reactions to obtain heterocyclic derivatives is very attractive. Recently, He and colleagues described by the first time the use of C–H/N–H oxidative cross-coupling/cyclization reactions starting with 2-aminopyridine (**13**), an alkyne (**46**), and a transition metal (Ag) affording 2-aryl substituted imidazo­[1,2-*a*]­pyridines (Figure 16B). This protocol was useful with varied terminal alkynes and for the synthesis of the antiulcer drug Zolimidine (**10**) in two steps (Figure 16A). The reaction proceeds through a catalytic cycle in which the most important steps are the oxidative addition followed by the reductive elimination step, which generates at the end of the catalytic cycle the desired imidazo­[1,2-*a*]­pyridine nucleus (**50**) (Figure 16B).^22,52^

**Figure 16:**
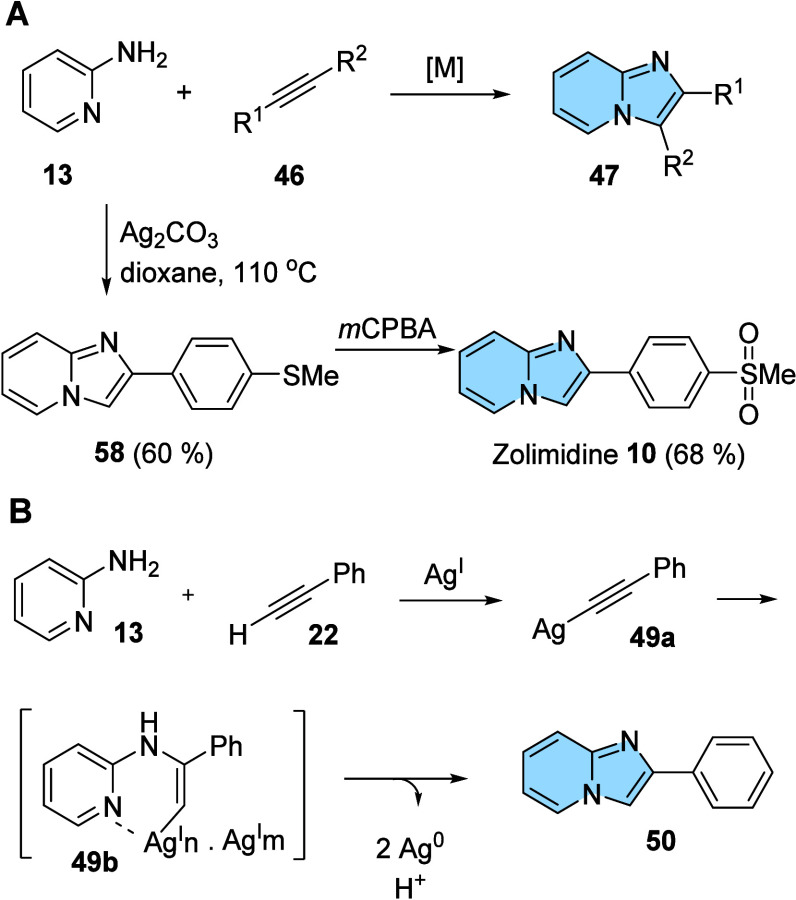
(A) General scheme of the synthesis of imidazo­[1,2-*a*]­pyridines (**47**) from oxidative couplings. (B) Proposed mechanism for the formation of 3-aryl-imidazo­[1,2-*a*]­pyridines (**50**).^22^

Instead of using expansive silver as a metal for C–H/N–H oxidative cross-coupling/cyclization reactions, some protocols were adapted to cooper. Dwivedi et al. developed a protocol for the synthesis of imidazo­[1,2-*a*]­pyridines heterosubstituted at the C3 position (**52**) through the reaction of disubstituted alkynes (**51**) with high electron density and 2-aminopyridine (**13**) under copper catalysis and atmospheric oxygen. Among the copper catalysts evaluated, Cu­(OTf)_2_ showed the highest conversion rate of the reactants and the reaction using acetonitrile as solvent was more efficient than DMF, DMSO, and toluene (Figure 17A). The protocol developed by the researchers makes it possible to obtain heterosubstituted C3 imidazo­[1,2-*a*]­pyridine derivatives (**52**) that are of great interest in medicinal chemistry for the development of bioactive molecules.^53^

**Figure 17:**
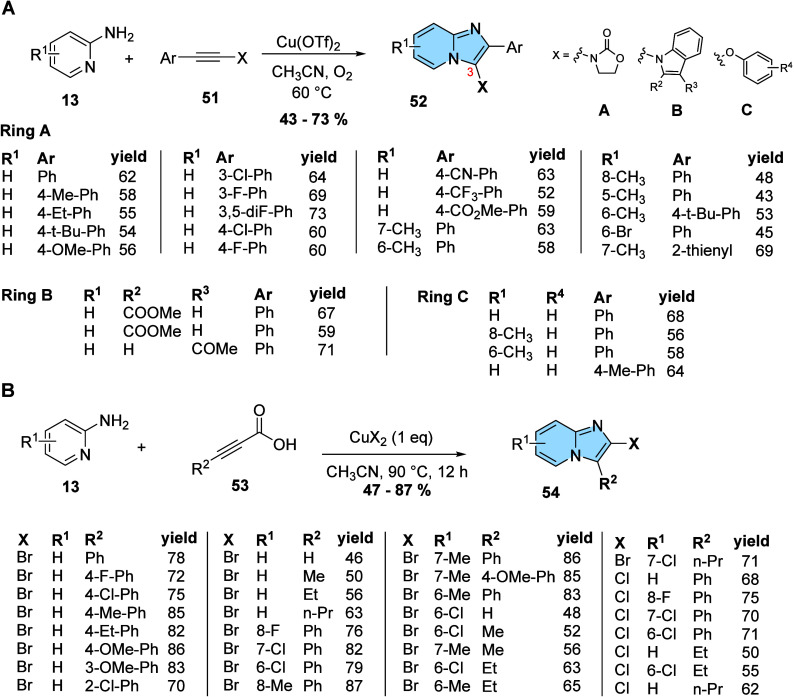
(A) Synthesis of heterosubstituted C3 imidazo­[1,2-*a*]­pyridine derivatives (**52**). (B) Synthesis of 2-halo-imidazo­[1,2-*a*]­pyridine derivatives (**54**).

Also using Cu^2+^ promoted coupling reactions, Liu and his group described the synthesis of novel 2-halo-imidazo­[1,2-*a*]­pyridine derivatives (**54**) from 2-aminopyridine (**13**) and alkynes (**53**) via sequential decarboxylative halogenation and oxidative diamination. The methodology proved to be very effective regardless of the nature of R^2^ (hydrogen, alkyl, or aryl groups). The use of CuCl_2_ or CuBr_2_ as a catalyst led to the formation of distinct products (*X* = Cl or Br), being very useful for possible derivatizations in the 2-halo-imidazo­[1,2-*a*]­pyridine scaffold, like Suzuki cross-coupling reactions, described by the authors themselves. The protocol described by the group showed good tolerance for several substituents in R_^1^_ in 2-aminopyridine and R^2^ in alkyne, resulting in moderate and optimal yields, even on a large scale (Figure 17B).^54^

On the other hand, Samanta and Bera developed a protocol for the synthesis of 2-aryl-imidazo­[1,2-*a*]­pyridines (**55a**) and 2-aryl-3-*S*-methyl-imidazo­[1,2-*a*]­pyridines (**55b**) through oxidative coupling reactions without the use of metallic catalysts (Figure 18). The authors developed an efficient molecular iodine-catalyzed protocol from 2-aminopyridine (**13**) and phenylacetylenes (**22**) in DMSO with satisfactory yields. The methodology also promotes *S*-methylation at the C3 position by increasing the temperature.^55^

**Figure 18:**
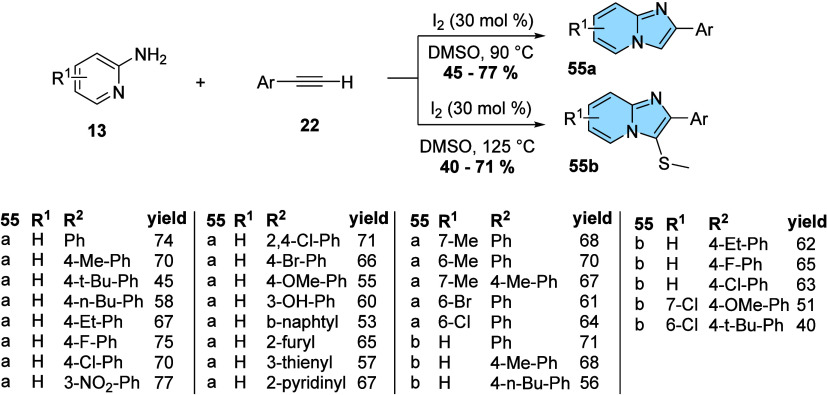
Iodine-catalyzed synthesis of 2-aryl-imidazo­[1,2-*a*]­pyridines and 2-aryl-3-*S*-methyl-imidazo­[1,2-*a*]­pyridines.

### 2.5 Intramolecular Cyclization Reactions

*N*-substituted derivatives of 2-aminopyridine (**56a**–**b**) are also substrate to obtain the imidazo­[1,2-*a*]­pyridine scaffold (**57**) through intramolecular cyclization reactions (Figure 19). From classical reactions, *N*-vinyl-aminopyridines and *N*-propargyl-aminopyridines can be synthesized and subsequently subjected to cyclization conditions, generating the desired imidazo­[1,2-*a*]­pyridines.^56–59^

**Figure 19:**
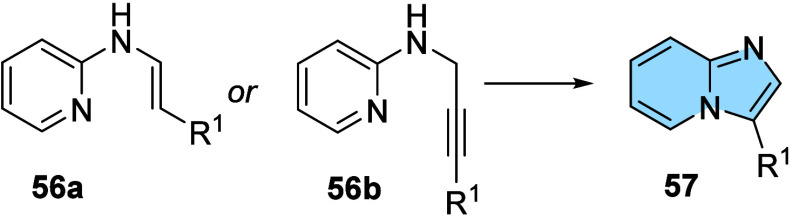
General scheme of the synthesis of imidazo­[1,2-*a*]­pyridines from intramolecular cyclization.

The use of *N*-vinyl- and *N*-propargyl-aminopyridines is common to intramolecular cyclization reactions to obtain imidazo­[1,2-*a*]­pyridines with or without catalysts. Cacchi and colleagues developed a new copper-catalyzed methodology to obtain imidazo­[1,2-*a*]­pyridine derivatives substituted at different positions from *N*-(2-pyridinyl)-enaminones (**58**). This new method allowed the introduction of acylated substituents at the C3 position of the imidazo­[1,2-*a*]­pyridine nucleus in a simple, efficient, and alternative way to available methodologies. The proposed mechanism begins with the formation of complex **58a** through the reaction of **58** with Cu­(I) under basic conditions, and the next intramolecular attack of the pyridine nitrogen to copper promotes **58b**. Subsequently, through a protonation reaction followed by a rearomatization/tautomerization process, **58b** converted to **58c** and finally, **59** via reductive elimination of CuH.^60^ Several ligands, solvents, and bases were evaluated, and the condition with CuI, Li_2_CO_3_, 1,10-phenanthroline, and DMF was the most efficient, presenting yields of 46–96% and reaction times that varied from 2 to 24 h depending on the electronic nature of the substituents (Figure 20A).^60^

**Figure 20:**
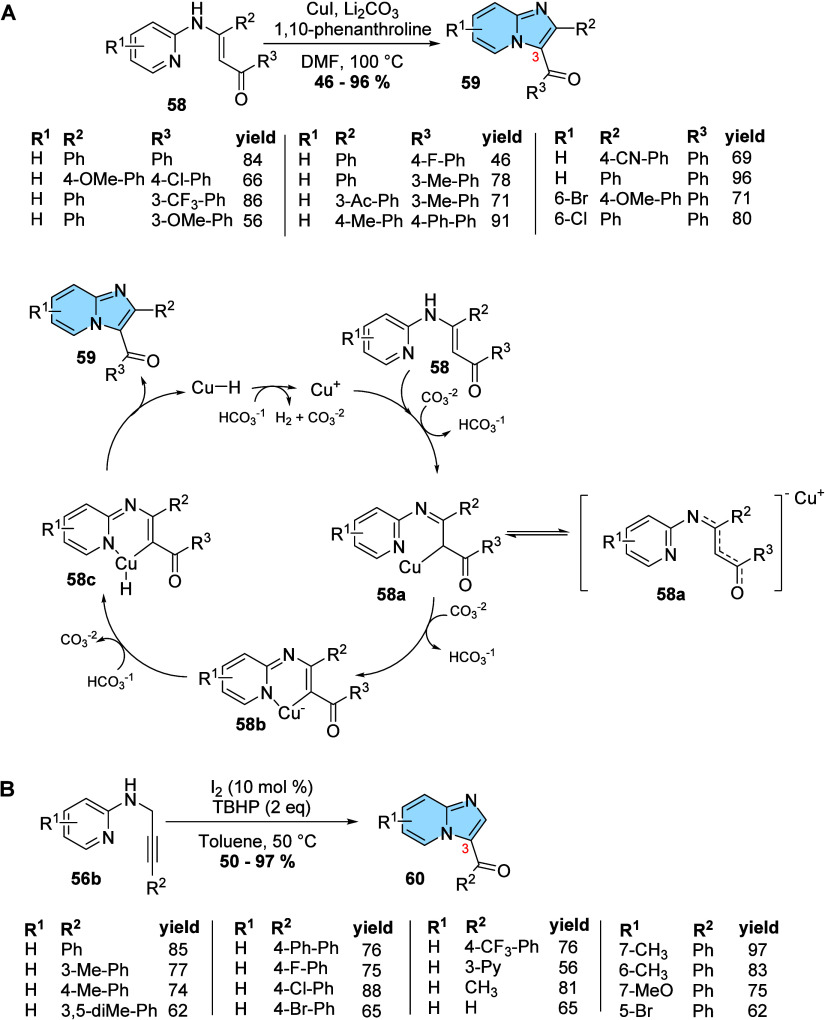
Intramolecular synthesis of imidazo­[1,2-*a*]­pyridines with *N*-vinyl- and *N*-propargyl-aminopyridines. (A) Synthesis and proposed mechanism of 3-acyl-imidazo­[1,2-*a*]­pyridine derivatives using CuI as catalyst (**59**).^60^ (B) Synthesis of 3-acyl-imidazo­[1,2-*a*]­pyridines (**60**) using I_2_ as catalyst.

A green methodology for obtaining 3-acyl-imidazo­[1,2-*a*]­pyridine derivatives (**60**) was proposed by He and his research group. The reaction proceeds using molecular iodine as a catalyst and *tert*-butyl-hydroperoxide (TBHP) as an oxidizing agent and presents a wide range of substrates in R^2^ such as hydrogen, alkyl, aryl, and heteroaromatic groups (Figure 20B). The reaction remains reproducible on large scales, without transition metals; has a simple execution protocol; and provides a wide variety of substitutions. These characteristics make this methodology attractive in the development of new molecules with biological activity.^61^

Conversely, Lee and co-workers described a metal-free method for obtaining benzo­[4,5]­imidazo­[1,2-*a*]­pyridines (**62**). This reaction occurs from the intramolecular cyclization of *N*-phenyl-2-aminopyridine (**61**) in the presence of chloramine-T and hexafluoroisopropanol (HFIP) at room temperature, showing excellent yields (32–93%) at very low reaction times (30 min), even when performed on a large scale (Figure 21).^62^

**Figure 21:**
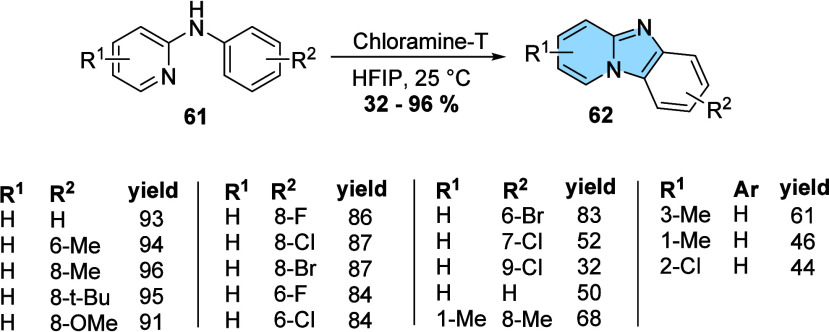
Synthesis of benzo­[4,5]­imidazo­[1,2-*a*]­pyridines (**62**).

### 2.6 Synthesis of Imidazo­[1,2-*a*]­pyridines under Green Conditions

In recent years, the application of sustainable reactions in organic chemistry has increased because of environmental concerns. Conventional organic chemistry methods often involve higher temperatures and the use of toxic organic oxidants, ligands, and solvents that lead to unwanted chemical waste. Additionally, the traditional synthesis has expensive chemical methods and is time-consuming.^63,64^

To address these problems, many recent studies have used green conditions in traditional chemical methods. For example, the synthesis of imidazo­[1,2-*a*]­pyridine has advanced using greener protocols in traditional reactions, such as condensation and multicomponent reactions, through green solvents, catalyst-free or solvent-free systems, photochemical reactions, and metal-free catalysis.^65,66^

Some green protocols have been applied to the synthesis of imidazo­[1,2-*a*]­pyridines. While chapters 2.1 and 2.2 covered conventional condensation and MCR reactions, Figure 22 shows modifications of these reactions using greener solvents.

**Figure 22:**
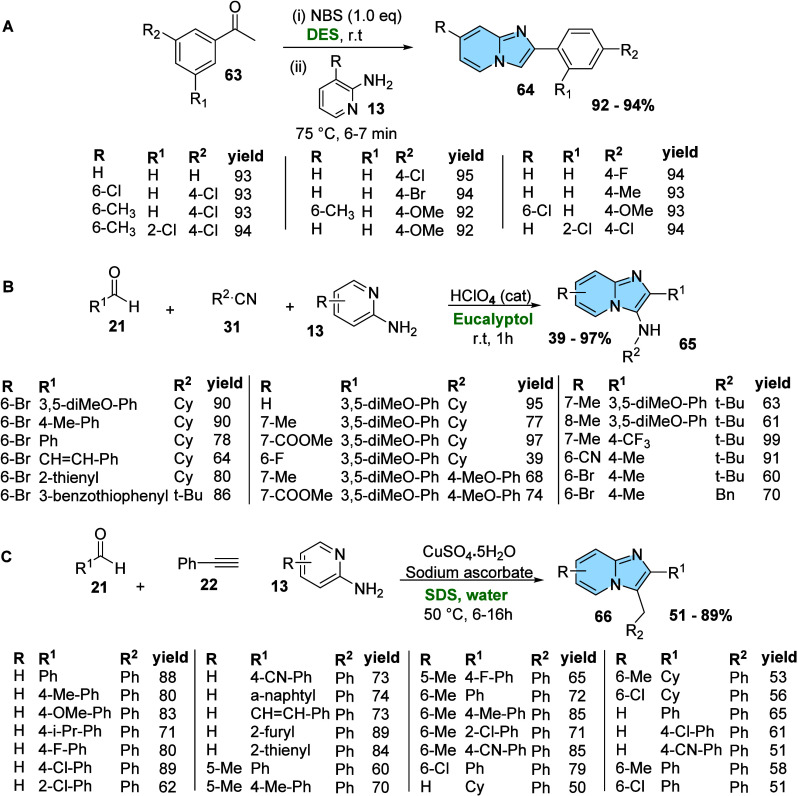
Green solvent in traditional methodologies of imidazo­[1,2-*a*]­pyridine synthesis. (A) One-pot condensation reaction of NBS, acetophenones, and 2-aminopyridines in deep eutectic solvent. (B) GBB reaction of aldehyde, 2-aminopyridines, isonitrile under sustainable conditions using eucalyptol as a green solvent. (C) Reaction of 2-aminopyridine, aldehyde, and alkyne through A^3^-coupling reaction.

The adaptation of the one-pot condensation reaction described by Shaikh and Kasim^67^ (Figure 22A) uses deep eutectic solvent (DES) as a good alternative to synthesize substituted imidazo­[1,2-*a*]­pyridine (**64**) with excellent yields in a few minutes, mild reaction conditions, and economy. Indeed, DESs are increasingly recognized as a new class of ionic liquid (IL) analogues, sharing many similar properties, such as being a green solvent. Although the terms DES and IL are often used interchangeably, they represent distinct solvent types. DESs are eutectic mixtures of Lewis or Bro̷nsted acids and bases containing various anionic and/or cationic species.^68^

Following the same concept, El Qami and colleagues^69^ reported an interesting example of a GBB reaction carried out under sustainable conditions (Figure 22B). Some green solvents, such as cyclopentyl methyl ether (CPME), 2-methyltetrahydrofuran (MeTHF), limonene, and eucalyptol, were tested in the presence of a catalytic amount of perchloric acid (HClO_4_). The procedure was tested at room temperature and under microwave irradiation, and eucalyptol showed the best results under both conditions. It is worth noting that eucalyptol occurs naturally in numerous plant extracts, particularly in the leaves and branches of eucalyptus trees. Because eucalyptus is widely cultivated for its fast growth (reaching maturity in about ten years), the use of eucalyptol in organic synthesis could also help reduce nonrecycled waste generated by the paper industry.

Among the various environmentally friendly solvents, water continues to be regarded as the solvent of choice.^70^ However, because many organic reagents and metal catalysts have limited solubility in water, a practical solution is to employ surfactants that promote the spontaneous formation of micellar aqueous systems. These micellar aggregates, generated through hydrophobic interactions, act as nanoreactors, creating localized environments with increased reactant concentrations.^71^ As a result, reaction rates and solvation effects are enhanced, which can improve chemo-, regio-, and stereoselectivity in the final products.^72^

Bhutia et al.^73^ developed an efficient and green synthetic protocol to the synthesis of imidazo­[1,2-*a*]­pyridines (**66**) by Cu­(II)–ascorbate-catalyzed domino A^3^-coupling in aqueous micellar media. This protocol used aldehyde MCR of aldehyde (**21**), alkyne (**22**), and aminopyridine (**13**) with copper catalyst (A^3^-coupling reaction) using water in the presence of sodium dodecyl sulfate (SDS) as the surfactant (Figure 22C). An *in situ* generated Cu­(II)/Cu­(I) catalytic system, formed by the reaction of CuSO_4_ with sodium ascorbate, efficiently promotes a 5-exo-dig cycloisomerization of alkynes with the condensation products of 2-aminopyridines (**13**) and aromatic aldehydes (**21**). This method performed effectively across a wide range of aliphatic, aromatic, and heteroaromatic aldehydes, showing good tolerance toward both electron-withdrawing and electron-donating substituents on 2-aminopyridine and benzaldehyde derivatives.

The use of solvent-free or catalyst-free is another strategy to focus on green conditions in organic synthesis (Figure 23). Yadav developed an MCR for the regioselective preparation of imidazo­[1,2-*a*]­pyridine (**69**) through a reaction of 2-aminopyridines (**13**), phenylglyoxal (**67**), and 4-hydroxy-6-methyl-2H-pyran (**68**) in ethanol under catalyst-free reaction conditions (Figure 23A). The authors demonstrated that the methodology is extremely versatile and robust, providing products in excellent yields even when donor or electron acceptor substituents are present in pyridine or phenyl ring.^74^

**Figure 23:**
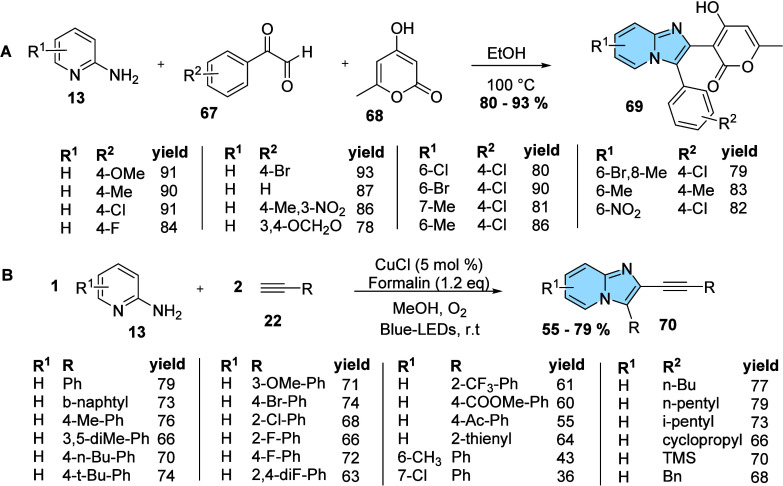
Catalytic modifications of imidazo­[1,2-*a*]­pyridine derivatives under green conditions. (A) Multicomponent synthesis of imidazo­[1,2-*a*]­pyridines (**69**) in ethanol under catalyst-free conditions. (B) Multicomponent synthesis of imidazo­[1,2-*a*]­pyridines (**70**) in photoredox catalysis.

Instead of free-catalyst reactions, recent studies have also focused on photochemical methods using photocatalysts or photochemical conditions for the regioselective synthesis of imidazo­[1,2-*a*]­pyridine as a green chemistry alternative.^65^ Charpe, Gupta, and Hwang^64^ developed a photoredox catalysis method, a visible-light-mediated copper­(I) chloride-catalyzed synthesis of imidazo­[1,2-*a*]­pyridines (**70**), using molecular oxygen as a green oxidant (Figure 23B). Charpe and colleagues synthesized 28 compounds in good yields. Even if the procedure takes longer than the previous ones, this photochemical method is low-energy and cost-effective, operates under greener conditions than thermal processes, and usually produces only water and CO_2_ as benign byproducts.

## 3 Imidazo[1,2-*a*]pyridines in Medicinal Chemistry

In Section 1, we discuss the relevance of imidazo­[1,2-*a*]­pyridines in studies involving biological systems. To identify the main areas of interest in Medicinal Chemistry, we searched the Scopus database for the period 2017–2025 using the term imidazo­[1,2-*a*]­pyridines in article titles, abstracts, or keywords, while restricting the search to leading Medicinal Chemistry journals (Journal of Medicinal Chemistry, European Journal of Medicinal Chemistry, Bioorganic and Medicinal Chemistry, Bioorganic Chemistry, Bioorganic and Medicinal Chemistry Letters, Rsc Medicinal Chemistry, Chemmedchem, Pharmaceuticals, Future Medicinal Chemistry, ACS Chemical Neuroscience, Chemical Biology and Drug Design, ACS Medicinal Chemistry Letters).

During the period covered in this review, 138 articles were published in these journals. The primary subject areas were cancer (*n* = 53; 38%), central nervous system disorders (*n* = 28; 20%), microbiology (*n* = 15; 11%), and parasitology (*n* = 10; 7%). Consequently, these four areas were selected for the following sections on the Medicinal Chemistry of imidazo­[1,2-*a*]­pyridines.

### 3.1 Imidazo­[1,2-*a*]­pyridines Designed for Cancer Targets

Cancer is a generic term that refers to a set of more than 100 types of genetic diseases, which are similar due to disordered cell growth, a consequence of multiple mutations accumulated during an individual’s life. Among the capabilities acquired by most, if not all, tumor types are self-sufficiency of growth signals, insensitivity to antigrowth signaling, inhibition of apoptosis, support of angiogenesis, tissue invasion and metastasis, and resistance to cellular senescence.^75^ Thus, the discovery of new antitumor drugs, more effective and with different mechanisms of action, is increasingly necessary.^76^

Microtubules, composed of heterodimers of α-tubulin and β-tubulin, are very important in the process of cell mitosis, during which the duplicated chromosomes of a cell separate into two identical sets before cleavage into two daughter cells. Thus, microtubules are strategic therapeutic targets for anticancer therapy.^77,78^

Imidazo­[1,2-*a*]­pyridine compounds have been reported as tubulin inhibitors (e.g., compound **71a**). In addition, imidazo­[1,2-*a*]­pyridine–oxadiazole hybrids (e.g., compound **72**) have been developed, inspired by earlier studies that also identified oxadiazoles as exhibiting antitubulin activity (e.g., compound **71b**).^79^ From the hybrid compounds, **72** stood out with a tubulin IC_50_ of 3.45 μM (Figure 24A), demonstrating a potent antiproliferative activity in all cell lines evaluated (A549: IC_50_ = 2.8 ± 0.02 μM; PC-3: IC_50_ = 3.5 ± 0.12 μM; DU-145: IC_50_ = 4.5 ± 0.2 μM) and selectivity index (SI) of at least 19 times when compared to normal human embryonic kidney cells (HEK-293). Immunohistochemical assay performed on A549 cells with compound **72** corroborated with its mechanism of action, showing efficient interruption of the organization of microtubules. Preliminary SAR for this series revealed that electron-donating groups (4-methoxy, 3,4-dimethoxy, 3,4,5-trimethoxy, and 4-*N,N*-dimethyl) on the phenyl ring of 1,3,4-oxadiazole enhanced bioactivity compared to unsubstituted counterparts. Conversely, electron-withdrawing groups (4-chloro, 4-bromo, and 2-iodo) reduced the cytotoxicity potencies. Regarding tubulin inhibition, maybe this SAR is not a surprise, since polyalkoxyphenyl groups are also related as tubulin pharmacophores. Molecular modeling showed that compound **72** fits well at the α/β-tubulin interface, forming key interactions, including hydrogen bonds via its oxadiazole nitrogen with Asn249 and Ala250, while the 3,4,5-trimethoxyphenyl ring establishes a π–cation interaction with Lys254 and hydrophobic contacts with several residues such as Ala250, Val181, Tyr224, and Met259.

**Figure 24:**
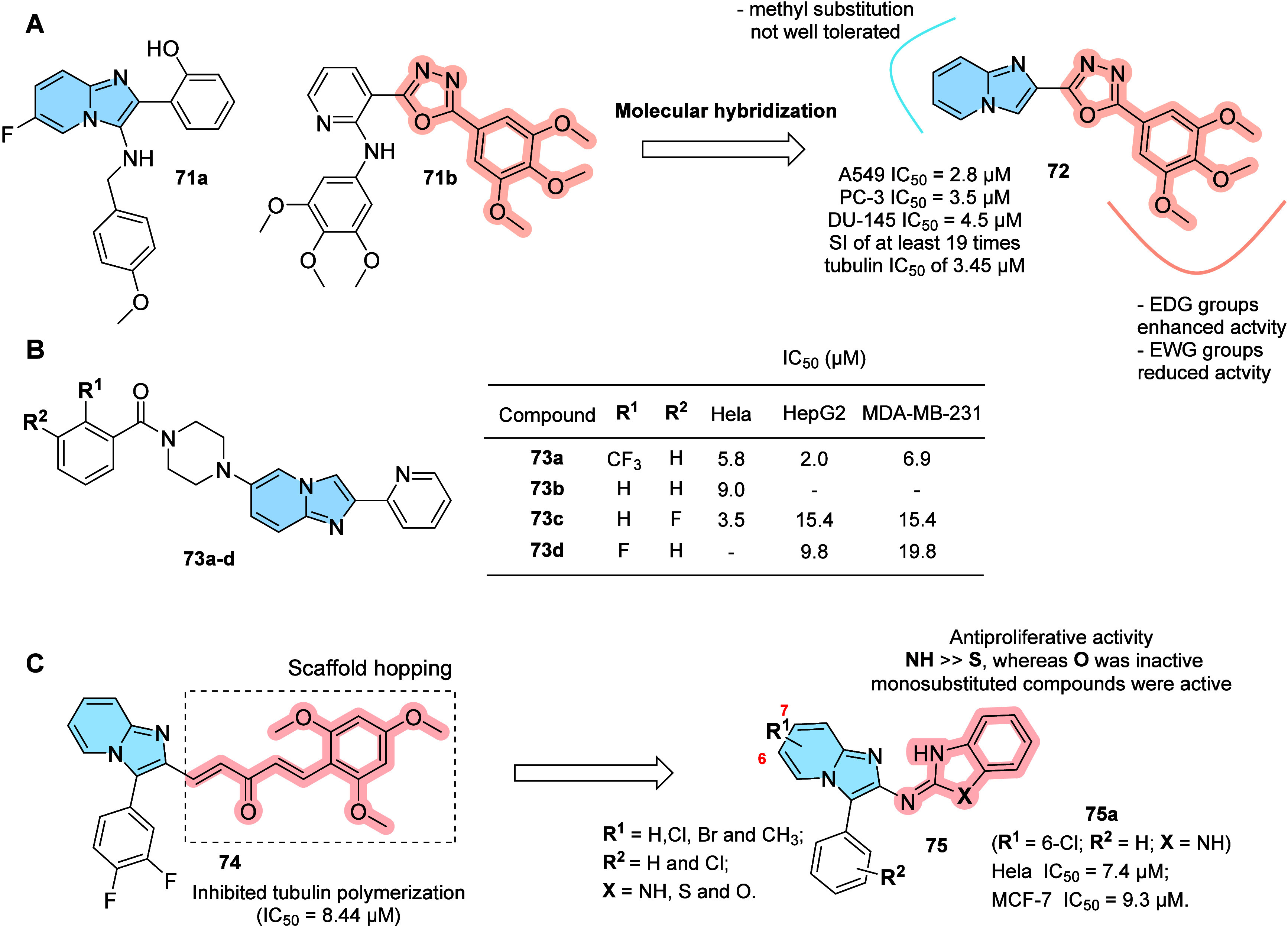
Imidazo­[1,2-*a*]­pyridine derivatives designed as tubulin inhibitors: (A) Imidazo­[1,2-*a*]­pyridine-oxadiazole (**72**) hybrid derivatives designed from tubuline inhibitors **70** and **71**, exhibiting anticancer activity via a G2/M cell cycle arrest and the induction of apoptosis. (B) Imidazo­[1,2-*a*]­pyridine derivatives (**73a–d**) with antiproliferative activity and possible dual inhibition mode of tubulin and estrogen receptor. (C) Compound **75a**, an imidazo­[1,2-*a*]­pyridine–benzoheterobicyclic hybrid, inhibited cancer cell proliferation via tubulin.

While compound **71b** retains the 3,4,5-trimethoxyphenyl pharmacophore, the role of the imidazo­[1,2-*a*]­pyridine core in tubulin inhibition was demonstrated by the 2-(pyridin-2-yl)­imidazo­[1,2-*a*]­pyridine series (**73**) and imidazo­[1,2-*a*]­pyridine-benzoheterobicyclic hybrids (**75**), which lacks the former pharmacophore (Figure 24B).^80,81^ Significant cytotoxic activities were observed against breast (MDA-MB-231), cervical (HeLa), and liver (HepG2) cell lines, especially compounds **73a**, **73b**, and **73c** (IC_50_ = 5.8 μM, 9.0 μM and 3.5 μM against HeLa respectively); **73a**, **73c**, and **73d** (IC_50_ = 2.0 μM, 15.4 μM and 9.8 μM against HepG2 respectively); and **73a**, **73c**, and **73d** (IC_50_ = 6.9 μM, 15.4 μM and 19.8 μM against MDA-MB-231 respectively), with the fluorine-substituted derivatives (**73a, 73c**, and **73d**) displaying the best activity against MDA-MB-231, HeLa, and HepG2 cell lines, suggesting that the polar hydrophilicity should play a key role in determining these potencies. Additionally, molecular docking showed that these compounds interact within tubulin in the colchicine binding site (CBS), located at the interface between the α and β subunits of tubulin heterodimers, as well as with the estrogen receptor active site, suggesting that they can act as multitarget compounds.

Sarkar et al. reported a series of *N*-linked imidazo­[1,2-*a*]­pyridine–benzoheterobicyclic hybrids based on **74** ^82^ as colchicine-site agents. Compound **75a** (R^1^ = 6-Cl; R^2^ = H; *X* = NH, Figure 24C) showed notable antiproliferative activity in HeLa cells, with an IC_5__0_ of 7.4 μM, inducing ∼40% microtubule depolymerization and inhibiting tubulin polymerization by 57% at 30 μM. SAR analysis showed that monohalogenated compounds retained activity, whereas disubstituted analogs were inactive; methylation at positions 6 or 7 of the imidazo­[1,2-*a*]­pyridine nucleus suppressed activity. These results support the potential of this scaffold to target the colchicine binding site and warrant further studies to assess pharmacological safety and chemical stability.^81^

Histone deacetylases (HDACs) play an important role in epigenetic transcriptional regulation by catalyzing the deacetylation of ε-*N*-acetyl-lysine residues of histone and nonhistone proteins. HDACs are overexpressed in several types of cancer, affecting several oncogenes and tumor-suppressor genes. Thus, the use of HDAC inhibitors has emerged as a promising strategy for anticancer treatment.^83^

HDAC inhibitors have a known pharmacophore model composed of three regions: zinc bind group (ZBG), a linker, and a CAP group. In this context, imidazo­[1,2-*a*]­pyridine group has been described recently in three series (**76a**–**c**) as an interesting CAP group. The first series designed as HDAC inhibitors was obtained from the multicomponent Groebke–Blackburn–Bienayme (GBB) reaction.^84^ The series exhibited IC_5__0_ values ranging from 0.13 to 3.33 μM for HDAC1 and from 0.050 to 1.33 μM for HDAC6, showing a greater selectivity for HDAC6. This selectivity may reduce side effects, while additionally, HDAC6 is involved in regulating nonhistone proteins such as α-tubulin and Hsp90, both of which are associated with neoplastic processes. The imidazo­[1,2-*a*]­pyridine derivative **76a** (Figure 25A) stood out as the most selective HDAC inhibitor (selectivity index = 38; HDAC1 IC_50_ = 2.20 μM, and HDAC6 IC_50_ = 0.058 μM), which was corroborated by the Western blot assay. Cocrystallization experiments showed compound **76a** linked to the second catalytic zinc-finger domain of *Danio rerio* HDAC6 in a monodentate binding mode with Zn^2+^ (Figure 25B).^84^ Interestingly, different from other aromatic capping groups, the imidazo­[1,2-*a*]­pyridine ring is turned to Phe643, instead of the normally observed binding to Pro464 (Figure 25B). This unique feature can be further explored in the design of HDAC6-selective inhibitors, as the occupancy of this region is associated with HDAC6 substrate recognition.

**Figure 25:**
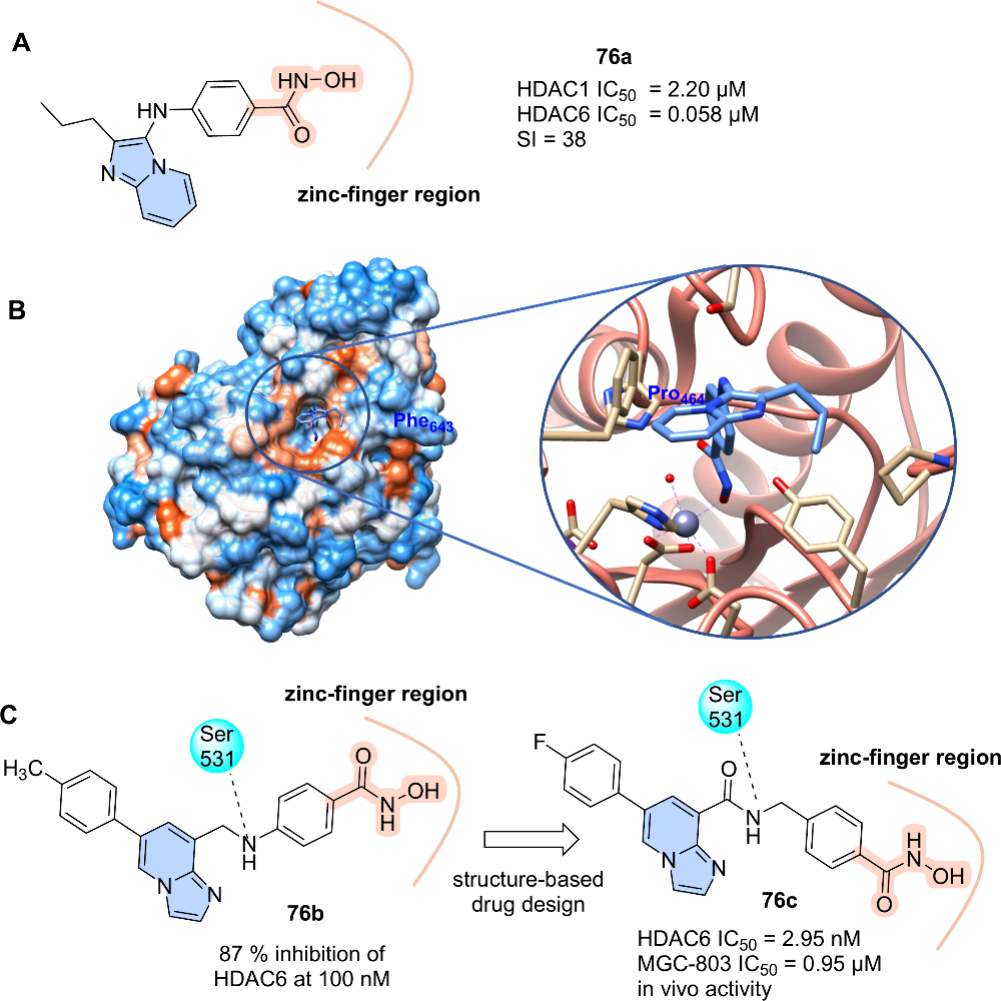
(A) Imidazo­[1,2-*a*]­pyridine derivatives (76), selective inhibitors of HDAC6. (B) Binding mode with HDAC6 (PDB 6CGP). (C) Selective HDAC6 inhibitors of imidazo­[1,2-*a*]­pyridine class (**76a–b**).

Indeed, recently, two new series of imidazo­[1,2-*a*]­pyridines (represented by compounds **76b** and **76c**) were described as HDAC inhibitors, in which this nucleus acts as a CAP group for both series. Compound **76b** (87% inhibition of HDAC6 at 100 nM) was identified from a screening of a chemical library consisting of approximately 500 nitrogen-containing heterocycle compounds in HDAC6, followed by molecular optimizations (Figure 25C). Interestingly, although compound **76b** was discovered through library screening, a quick comparison with compound **76a** reveals classic medicinal chemistry modifications such as homologation and regioisosterism. Docking studies with **74a** demonstrated that hydroxamic acid (ZBG) formed a double-coordination chelate mode with Zn^2+^ at the zinc-finger region of the enzyme. At the same time, the phenyl group of the linker was in a hydrophobic/aromatic cavity composed of Phe583 and Phe643, forming a specific π–π stacking effect. The benzylamine spacer presented a significant influence on both inhibitory activity and selectivity due to interaction.^85^ Indeed, the nitrogen atom of the linker forms a key hydrogen bond with Ser531, which is a unique amino acid in HDAC6-CD2. Interactions with Ser531 (Ser568 in hHDAC6) in loop L2 contribute to the HDAC6 selectivity. All HDAC isoforms have aspartic acid in this position except for HDAC6 (Asp to Ser), HDAC10 (Asp to Ala), and HDAC11 (Asp to Asn).^86^

More recently, Yang and collaborators described structure–activity relationship studies driven by pharmacophore-based remodification and fragment-based design that led to novel imidazo­[1,2-*a*]­pyridine derivatives presenting a different substitution pattern.

The introduction of an amide moiety in place of C–N of the linker followed by a superior homologation of a methylene group into the linker, increasing its length or flexibility, originated potent HDAC6 inhibitor compounds with cardioprotective properties. Derivative **76c**, presenting a 4-fluorophenyl and a carboxamide group at positions 6 and 8, respectively, of imidazopyrine stood out as the most potent and promisor HDAC6 inhibitor (IC_50_ = 2.95 nM), with the highest cytotoxicity observed in the MGC-803 cell line (IC_50_ = 0.95 ± 0.03 μM) (Figure 25C). Additionally, derivative **76c** showed a dose-dependent inhibition of MGC-803 cells growth in xenograft models (even at 250 nM) and *in vivo* effective suppression of the tumor volume and mass increase. Furthermore, no significant changes were observed in the weight of mice nor myocardial damage after long-term administration.

Protein kinases (PTKs) specifically phosphorylate other proteins, leading to the activation of intracellular signal transduction pathways fundamental in multiple biological processes, including growth, differentiation, and apoptosis, but their dysregulation and overexpression are characteristic of tumor cells.^87^

Based on this approach, Fan’s group^88^ described the synthesis of imidazo­[1,2-*a*]­pyridine derivatives and their respective antiproliferative activities against five cell lines, including HCT-116, SK-HEP-1, MDA-MB-231, SNU638, and A549, as well as PI3Ka protein kinase. The results showed most derivatives presenting expressive antiproliferative activities, especially against HCT-116 and SNU638. Compounds **77a** and **77b** (Figure 26A) were the most active, especially **77a** with an IC_50_ value of 0.01 μM against HCT-116 and SNU638; an IC_50_ value of 0.04 μM against MDA-MB-231 and A549; and an IC_50_ value of 0.11 μM against HEP-1. Compounds **77a** and **77b** were also less cytotoxic against normal cells in HUVEC, with IC_50s_ at least 290 times higher than those for HCT-116 and MDA-MB-231 cells, indicating a good safety profile. Furthermore, the kinase inhibition activity assay indicated that compounds **77a** and **77b** showed very significant inhibitory activity against PI3Kα (0.5 and 3.8 μM, respectively) when compared to other PI3Ks isoforms (PI3Kβ, PI3Kγ, and PI3Kδ) (Figure 26A). The Western blot assay showed that compound **77a** reduced phospho-Akt (S473) levels in a dose-dependent manner, indicating that this compound is inhibiting PI3K intracellular. Molecular docking studies have elucidated the probable mode of interaction between compound **77a** and the PI3Kα binding site, as well as its main interactions, namely, three hydrogen bonds between the sulfonamide and the Val859 residue, interaction between the morpholine ring and residue Lys802, and between the nitrogen of the pyridine group and residue Ser854, in addition to hydrophobic interactions with residues Lys802, Tyr836, Ile848, Val851, and Ile932.

**Figure 26:**
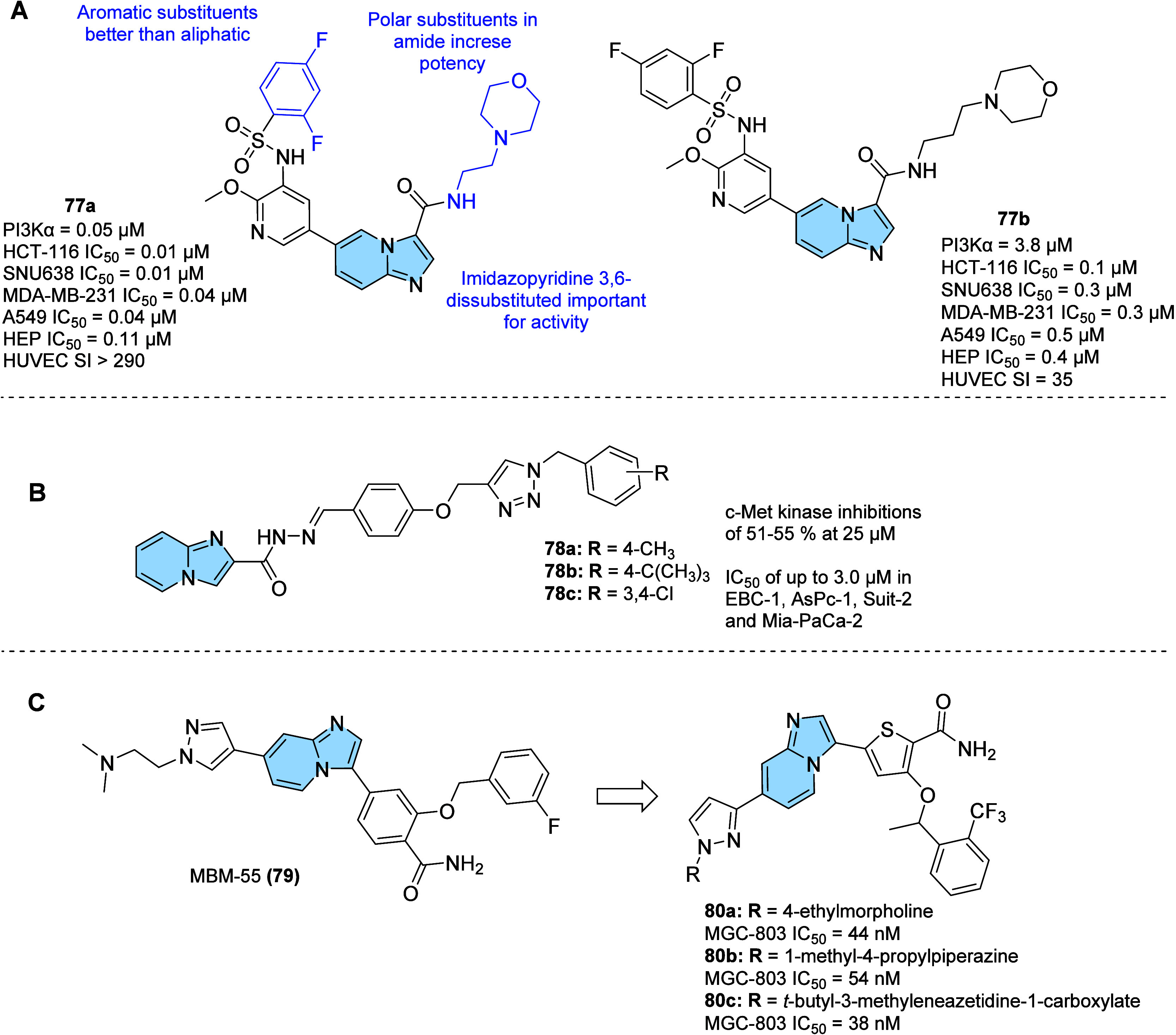
Imidazo­[1,2-*a*]­pyridine derivatives are designed as kinase inhibitors. (A) c-Met receptor tyrosine kinase inhibitors. (B) Imidazo­[1,2-*a*]­pyridine derivatives containing the 1,2,3-triazole (**78a–c**) c-Met kinase inhibitors. (C) Nek2 inhibitors.

Damghani et al.^89^ proposed a series of imidazo­[1,2-*a*]­pyridine derivatives containing the 1,2,3-triazole nucleus that showed an anticancer effect and potent inhibition of c-Met, a receptor that belongs to the receptor tyrosine kinase (RTK) family, which is expressed on the surfaces of various epithelial cells and plays essential roles in controlling critical cellular processes such as cell proliferation, survival, motility, and morphogenesis.

The synthesized compounds were evaluated *in vitro* for inhibitory activity against c-Met kinase using a homogeneous time-resolved fluorescence assay (HTRF). Compounds **78a**, **78b**, and **78c** (Figure 26B) showed the best inhibitions at 25 μM, with 55.3, 53.0, and 51.3%, respectively. The compounds also demonstrated *in vitro* antiproliferative activity against lung (EBC-1) and pancreas (AsPc-1, Suit-2, and Mia-PaCa-2) cancer cell lines, with IC_50s_ values of up to 3.0 μM. The Western blot assay indicated that the active derivatives significantly blocked c-Met phosphorylation, in addition to inhibiting cell growth in three-dimensional steroid cultures. Flow cytometry assay using Annexin V-FITC/PI demonstrated that derivatives **78a**–**c** induced apoptosis in AsPc-1 cells. These compounds were evaluated against a panel of 16 human kinases to study the kinase selectivity profile, which also demonstrated that the compounds could inhibit PDGFRA and FLT3 at 25 μM.

Following the kinase targeting scope, never-in-mitosis (NIMA)-related kinase 2 (Nek2) has been reported as a key regulator for cell cycle progression, being involved in the regulation of cell cycle checkpoint, cell division, response to DNA damage, and cellular apoptosis, representing an anticancer target with great potential. To explore this target, a series of imidazo­[1,2-*a*]­pyridine compounds^90^ were designed as potential inhibitors of Nek2, based on a prototype MBM-55 (**77**) previously published by Wang’s group.^91^ After extensive synthetic work, the derivatives were evaluated regarding the inhibition of the proliferation of gastric cancer cell lines, including MGC-803 cells, where promising results were obtained, emphasizing compound **80c**, which showed an IC_50_ of 38 nM. Based on these results, some compounds, **80a** (MGC-803 IC_50_ = 44 nM), **80b** (MGC-803 IC_50_ = 54 nM), and **80c** (MGC-803 IC_50_ = 38 nM), were selected to be evaluated for their antiproliferative activity against hepatoma cells (Hep-3B, BEL-7402) and colon cancer cells (HCT-116), where they showed to be active with inhibition potency similar to the prototype used (Figure 26C).^90^

In the last two decades, many efforts in discovering new cancer therapies have been related to Aurora kinase inhibitors because of their implication during cell division. The Aurora kinases (A, B, and C) are threonine protein kinases important to mitosis regulation. Aurora B is a component of the chromosome passenger complex (CPC) with three subunits: inner centromere protein (INCENP), Survivin, and Borealin. This phenotype is responsible for the selective destabilization of incorrect attachments between microtubules and kinetochores.^92–94^

The overexpression of Aurora B in many different tumor cells suggests that many cancers could respond therapeutically to inhibitors of the Aurora kinases. Bearing in mind the importance of Aurora kinases, Juillet and co-workers synthesized benzosceptrins and oroidin in reference to the marine pyrrole-2-aminoimidazoles metabolites isolated from sponges as Aurora B inhibitors. The compounds were synthesized exploring the molecular diversity from the initial hit EL-228 (**81a**). Many studies were performed such as the analysis of kinase inhibition in different cell lines, structure–activity relationships, selectivity in the inhibition of Aurora B, antiproliferative profiles, and molecular docking. The compound CJ2–150 (**81b**) was identified as the most active compound with nanomolar IC_50_ inhibition and high selectivity for both Aurora A and B. ATP-competition assay and molecular docking revealed that **81b** is a non-ATP competitive inhibitor of Aurora B kinase, in a mixed-type inhibition (Figure 27A).^95^

**Figure 27:**
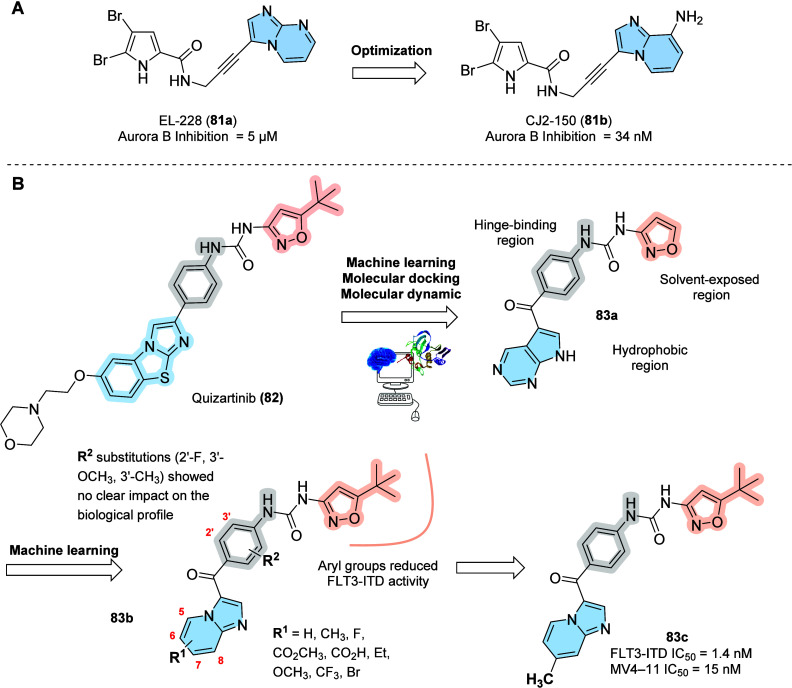
Imidazo­[1,2-*a*]­pyridine derivatives were designed as kinase inhibitors. (A) Allosteric Aurora B inhibitor; (B) FLT3 inhibitors.

Still within the kinase landscape, we highlight FLT3, a tyrosine kinase that regulates the survival and proliferation of hematopoietic cells, which is expressed in most patients with acute myeloid leukemia (AML). Constitutive mutations, including FLT3-ITD (25%) and point mutations in the kinase domain (5%), are associated with high relapse rates and reduced survival. Consequently, selective FLT3 inhibition is a promising strategy for FLT3-mutated AML.^96,97^ Huang et al. used a multivariate computational approach integrating machine learning, molecular docking, and molecular dynamics to identify 68 potential FLT3 inhibitors, starting from a Bemis-Murcko scaffold analysis on 1189 active molecules in the same target. This strategy led to compound **83a**, previewed as a potential inhibitor of FTL3-ITD kinase. Machine learning applied to scaffold modification on **83a** furnished imidazo­[1,2-*a*]­pyridine **83b** (R^1^ = H, R^2^ = H; FLT3-ITD IC_50_ = 2 nM; MV4–11 IC_50_ = 3.14 μM). Guided by these findings, they designed novel 4-(imidazo­[1,2-*a*]­pyridine-3-carbonyl)­phenylurea derivatives using medicinal chemistry strategies focusing on explorations in three specific regions (hydrophobic, hinge-binding, and solvent-exposed regions). The best compound (**83b**) showed excellent FLT3 potency (IC_50_ = 1.4 nM) and antiproliferative activities against MV4–11 cells (IC_50_ = 15 nM) (Figure 27B).^98^

New sulfenylated imidazo­[1,2-*a*]­pyridine derivatives were synthesized and evaluated by Chitrakar and collaborators^99^ for their *in vitro* antiproliferative activity against seven human cancer cell lines, such as breast (MDA-MB-231), liver (HepG2), cervical (HeLa), lung (A549), glioblastoma (U87MG), melanoma of the skin (SKMEL-28), and prostate (DU-145). Compounds **84a**, **84b**, and **84c** (Figure 28A) showed promising potent antiproliferative activity against HepG2 human liver cancer cells (**84a**: IC_50_ = 9.9 μM; **84b**: IC_50_ = 8.8 μM; **84c**: IC_50_ = 7.6 μM) (Figure 28A). Flow cytometry analysis revealed that these compounds were able to interrupt the cell cycle in the G2/M phase and induce apoptosis in the HepG2 cell line. The apoptotic effect of these compounds was confirmed by Hoechst staining, measurement of mitochondrial membrane potential (ΔΨm), and annexin V-FITC assay.

**Figure 28:**
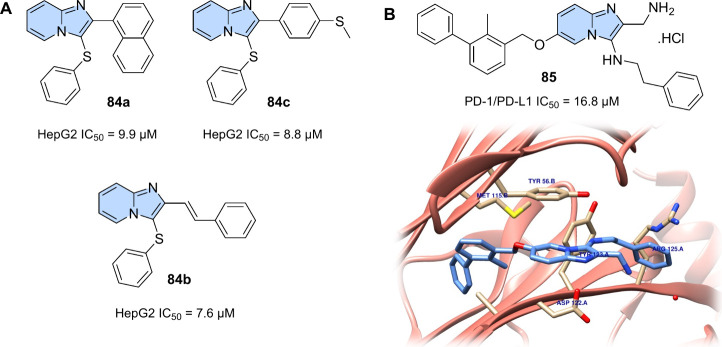
(A) Sulfenylated imidazo­[1,2-*a*]­pyridine derivatives (**84a–c**) with an antiproliferative activity through the G2/M arrest of the cell cycle and induction of apoptosis. (B) Imidazo­[1,2-*a*]­pyridine derivatives (**85**) PD-1/PD-L1 antagonists and their binding mode to the PD-L1 dimer (PDB 6R3K).

Immunotherapy is an important front in the treatment of cancer, since it acts directly on the body’s immune system, recognizing and eliminating tumor cells. In immunotherapy, drugs act at various immunological checkpoints of the body, recognized as several coinhibitory and costimulatory receptors and their ligands that act, under normal conditions, to protect the body against autoimmunity, allergies, and infectious diseases.^100^ Among them, the immune checkpoint block (ICB) of targets such as PD-1/PD-L1 (programmed cell death protein 1 and programmed cell death-ligand 1) and CTLA-4 (cytotoxic T-lymphocyte-associated antigen 4) stands out.^101^

In this context, Butera and colleagues^102^ proposed a series of compounds using an imidazopyridine scaffold to act as PD-1/PD-L1 antagonists. The compounds were synthesized through the multicomponent GBB reaction and subjected to binding NMR studies with PD-L1, and to a homogeneous time-resolved fluorescence (HTRF) assay, which showed that the compounds can disrupt the PD-1/PD-L1 complex formation and binding to PD-L1, as well as induce dimerization of PD-L1. The IC_50_ values obtained from the HTRF assay were in the range of 16.8–1.8 μM. A cocrystal of compound **85** (PD-1/PD-L1 IC_50_ = 16.8 μM) obtained with PD-L1 allowed the elucidation of the way in which these compounds interact with the target protein, showing the importance of imidazopyridine in making π-π (Tyr56) and cation-π bonds (Figure 28B).

Signal transducers and activators of transcription (STATs) are genes that can be classified by seven STAT proteins identified for mammalian cells: STAT1, 2, 3, 4, 5a, 5b, and 6. Under physiological conditions, these transcription factors regulate cell proliferation, differentiation, apoptosis, and immune and inflammatory responses. The STAT3 protein is involved in many cellular functions and play an important role in the development of multiple cancer types because of its activation during disease progression and metastasis in a variety of cancers.^103^

A potent and selective STAT3 small-molecule inhibitor has been discovered by Huang and colleagues derived from two series of substituted 2-phenylquinolines and 2-arylimidazo­[1,2-*a*]­pyridines. This compound was designed through structure-based drug discovery from STX-119 (**86**) and SH4–54 (**87**) structures, which are both selective STAT3 inhibitors. The research program started with molecular docking studies of selective STAT3 inhibitors (STX-119, MDA-MB-231, IC_50_ = 2.0 μM and SH4–54, MDA-MB-231 IC_50_ = 18.25 μM), which indicated that both have similar binding modes to an important domain of the protein. From these results, 33 substituted quinolines (series A) were synthesized and evaluated, showing promising results in the IC_50_ range of 5.3–50 μM in MDA-MB-231 cells (Figure 29). To obtain more potent compounds, attention was turned to optimizing the 2-phenylquinoline moiety since molecular modeling studies of these derivatives indicated that a decrease in the distance between C2-phenyl and C4-carbamoyl could increase the affinity of binding and consequently increase the inhibitory activity. Therefore, 27 imidazo­[1,2-*a*]­pyridines (series B) were synthesized and evaluated for cell growth inhibition (MDA-MB-231 IC_50_ = 0.7–20.2 μM, MGC-803 IC_50_ = 0.4–22.5 μM, A549 IC_50_ = 0.4–23.4 μM). Compound **89c**, with the structure of 2-phenylimidazo­[1,2-*a*]­pyridine, was identified as the most selective and potent inhibitor of STAT3 phosphorylation among series B compounds, also suppressing the subsequent signaling pathway. Additionally, cell growth inhibition, migration, and invasion were also observed in human triple negative breast cancer (TNBC) cells lines. These results clearly indicated that compound **89c** is a highly potent and selective STAT3 inhibitor (Figure 29).^104^

**Figure 29:**
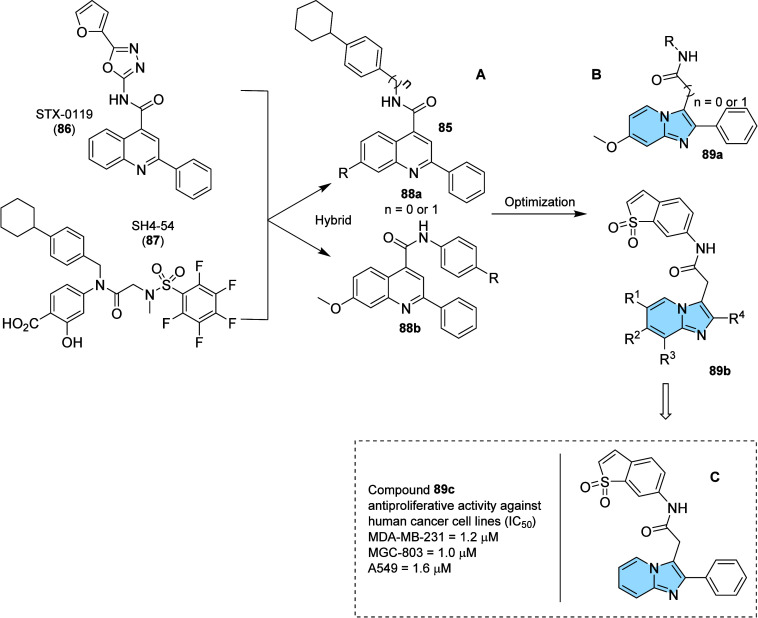
Design of new STAT3 inhibitors of quinoline derivatives (A) and imidazo­[1,2-*a*]­pyridines derivatives (B). Antiproliferative activity of the most active compound (C).

Other highlighted transcriptional regulators are those that mediate chromatin-dependent transcriptional signaling. Recently, the transcriptional coactivator ENL has been shown to play a crucial role in the survival of acute myeloid leukemia (AML). More specifically, the YEATS domain of ENL is thought to be responsible for mediating binding and colocalization in acetylated regions of chromatin.^105^ Inspired by the preliminary studies, Garnar-Wortzel and colleagues^106^ performed a high-throughput chemical screening. The best hits were subjected to HTRF assays, and ENL (YEATS)-HiBiT engagement was measured by ligand-induced luminescence, wherein amido-imidazopyridine compound **90** was identified as an inhibitor of the ENL YEATS domain (IC_50_ = 7 μM).

Based on this scaffold, structural modifications were proposed to establish a structure–activity relationship. These efforts led to the production of compound **91**, which proved to be a potent and selective inhibitor of the ENL/AF9 YEATS domain (IC_50_ = 25 nM). It was then observed that position *meta* on the phenyl ring in **91** would support long chains. Bearing this information in mind, they designed an ENL PROTAC (Proteolysis Targeting Chimeras) from the addition of a polyethylene glycol linker in *meta*, which binds to a thalidomide derived scaffold, the E3 recruiter moiety (Figure 30).

**Figure 30:**
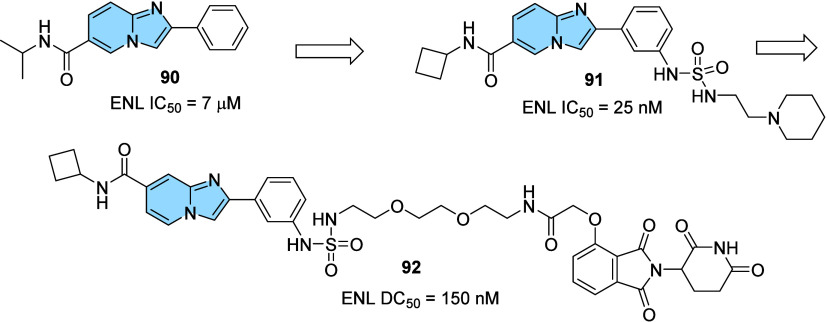
Discovery of the imidazo­[1,2-*a*]­pyridine derivative (**92**, SR-1114), the first-in-class ENL PROTAC.

Thus, compound **92** (SR-1114) was obtained, the first-in-class ENL PROTAC with a DC_50_ of 150 nM, effectively acting in the pharmacological interruption of ENL in AML cells. It is worth noting that the PROTAC technology represents an important and innovative approach based on an event-driven mode of action. By simultaneously binding to a target protein and an E3 ligase, PROTACs induce targeted protein degradation, thereby modulating protein levels. Several PROTACs are currently in clinical trials, with one example (ARV-471) already advancing to phase 3.^107^

Autotaxin (ATX) is an exoenzyme responsible for the release of bioactive signaling lipid lysophosphatidic acid (LPA). Recent studies show that the binding between LPA and ATX initiates LPA biological activity and facilitates the delivery of active LPA to its cell surface receptors, producing a cascade of cellular responses, including survival, migration, and proliferation. The ATX–LPA interaction is related to several diseases, for example, inflammation, fibrosis, autoimmune, cardiovascular diseases, and cancer.^108,109^

On the lookout for the potential antitumoral effect, Lei and collaborators designed and synthesized a novel imidazo­[1,2-*a*]­pyridine derivatives as potent autotaxin allosteric inhibitors (**95**) using a molecular hybridization^110^ strategy from ATX inhibitors PF-8380 (**93**) and GLPG-1690 (**94**) (Figure 31). Initially, the amino-thiazole group of GLPG-1690 was placed by 3,5-dichlorobenzyl carbamate moiety from PF-8380, while position 6 of imidazo­[1,2-*a*]­pyridine scaffold was substituted with phenyl groups, aiming at evaluating possible interactions with residues of Phe250 and Trp255. Furthermore, various substituted amines were introduced to the phenyl ring, which explored interactions within the ATX hydrophobic channel. All the compounds synthesized were interesting ATX inhibitors with IC_50_ values below 300 nM, highlighting compound **96** as the best ATX inhibitor with an IC_50_ value of 3.4 nM and the most potent antiproliferative compound in ATX abundant cancer cell lines, especially Hep-3B (0.58 μM) and RAW264.7 (0.63 μM).^108^

**Figure 31:**
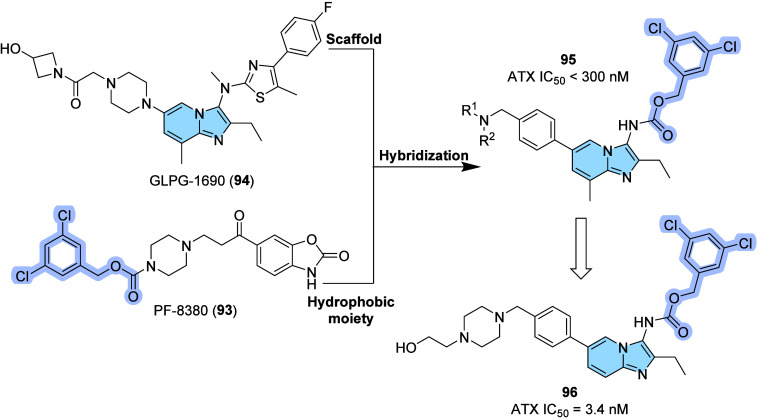
Structures of GLPG-1690 (**94**), PF-8380 (**93**), the general hybrid (**95**), and the most active compound **96** based on the FS-3 based ATX enzymatic assay.

### 3.2 Designed against Alzheimer’s Disease

Alzheimer’s disease (AD) is a chronic neurodegenerative disorder capable of causing progressive loss of memory and other functions. It is a complex disease with a multifactorial nature and undetermined origin. It is characterized by decreased neuronal cholinergic activity, deposition of β-amyloid (Aβ) peptide aggregates on the extracellular surface of neurons, intracellular deposits of insoluble neurofibrillary filaments composed of hyperphosphorylated forms of tau protein (PTau), neuroinflammation, dysfunction in metal homeostasis, and oxidative stress.^111^

Cholinesterase inhibitors are the main strategy used to treat cognitive and functional symptoms of AD. They act by blocking the catalytic site of the cholinesterase enzyme, which delays the metabolic degradation of acetylcholine (ACh) and, consequently, intensifies cholinergic transmission.^112–114^

Kwong and colleagues^115^ described the synthesis and *in vitro* and *in silico* anticholinesterase evaluation of imidazo­[1,2-*a*]­pyridines (Figure 32). The most active derivatives were compound **97a**, with an IC_50_ of 79 μM for acetylcholinesterase (AChE), and **97b**, with an IC_50_ of 65 μM for butyrylcholinesterase (BuChE). Molecular docking studies showed that compound **97a** interacts with both the catalytic site (CAS) and the peripheral site (PAS) of AChE, through π-π interactions between biphenyl and residues Trp279 and Tyr334 in PAS, and between the imidazo­[1,2-*a*]­pyridine ring and residues Trp84, Gly117, and Gly118 in CAS via hydrogen bonds and amide-π interactions. Regarding BuChE, it was observed that compound **97b** binds to CAS through the interaction between the imidazo­[1,2-*a*]­pyridine ring with Trp82 and His438, as well as via halogen–O interaction from subunit 3,4-dichloro-phenyl with Leu286.

**Figure 32:**
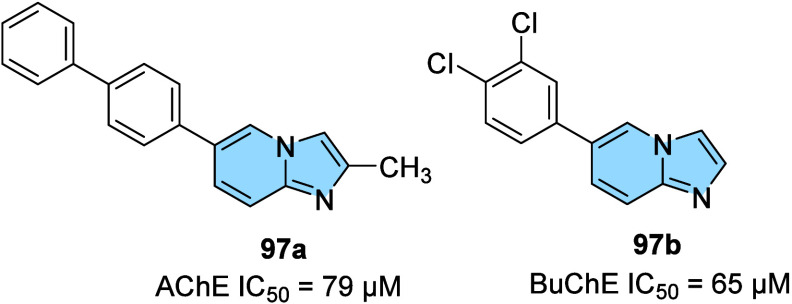
Imidazo­[1,2-*a*]­pyridine derivatives (**97a,b**) inhibitors of AChE and BuChE.

Secretase enzymes are responsible for processing amyloid precursor protein (APP) that forms Aβ peptides. APP can be transformed by the α-secretase enzyme and then by the γ-secretase enzyme in the nonamyloidogenic pathway. However, in the amyloidogenic pathway, APP is first cleaved by β-secretase (BACE) and then by γ-secretase, producing fibrillogenic Aβ peptides of 38 to 42 amino acids. Thus, inhibitors of these enzymes can reduce the formation of Aβ peptides, representing an important therapeutic strategy to prevent AD progression.^116^

In this context, a series of hybrid imidazopyridines-phthalimide derivatives designed as inhibitors of BACE1 were developed by Azimi and colleagues.^117^ The compounds were synthesized through the multicomponent GBB reaction, and derivatives **98a** and **98b** (Figure 33A) were the most active against BACE1 (IC_50_ = 2.84 and 5.93 μM, respectively). The SAR study indicated the important contribution of the cyclohexyl group to the increase of the inhibitory activity on BACE1, such as the presence of methyl in different positions of imidazopyridine. Through molecular docking studies, it was observed that derivatives containing the cyclohexyl group showed hydrogen-bonding interactions between the nitrogen atom of the imidazopyridines and the oxygen atom of the phenoxypropyl spacer with the Asp228 and Asp32 residues of the BACE1 active site, respectively.

**Figure 33:**
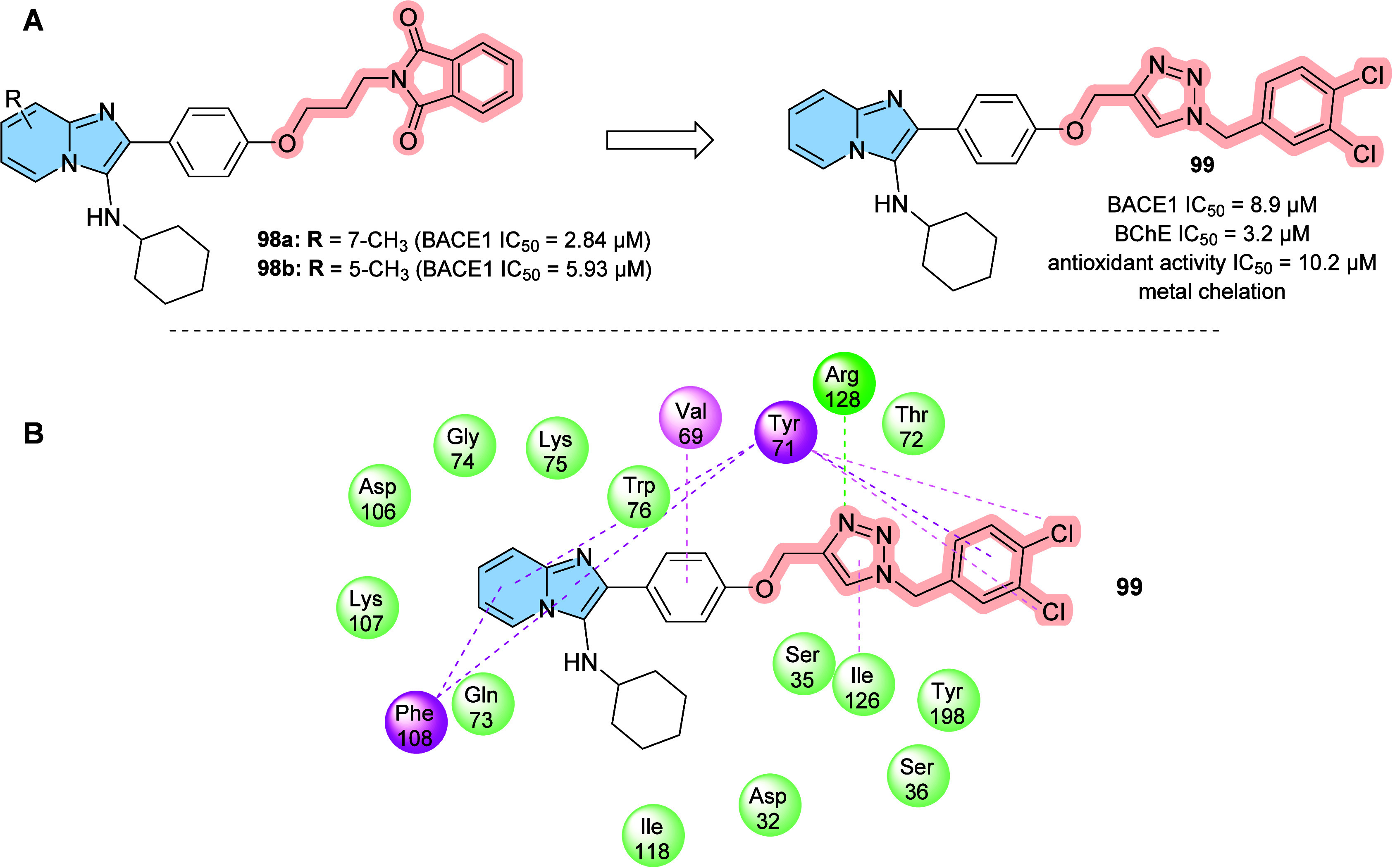
(A) Imidazo­[1,2-*a*]­pyridine derivatives (**98a-b** and **99**) inhibitors of BACE1 and BuChE. (B) Compound 99 2D model main interactions from molecular docking at the active site of BACE1.

Subsequently, based on the AChE inhibitory potential of the imidazo­[1,2-*a*]­pyridine derivatives described in the literature, the same group proposed a rational design of the *N*-cyclohexylimidazo­[1,2-*a*]­pyridin-3-amine scaffold as suitable for inhibiting BACE1 and AChE/BuChE. Substitutions of the phthalimide group for benzyl triazoles resulted in compounds with *in vitro* BACE1 and BuChE inhibition activity and antioxidant properties. Among the compounds, compound **99** (Figure 33A) stands out as one of the most active for BACE1 (IC_50_ = 8.9 μM) and for BuChE (IC_50_ = 3.2 μM), in addition to antioxidant activity (IC_50_ = 10.2 μM) and metal chelation potential. Molecular docking results performed in the active site of BACE1 indicated that imidazopyridine of compound **99** could make π–π stacking interactions with Tyr71, while the triazole ring makes H-bond interactions with Arg128. Additionally, other different π–π interactions between the compound and the residues Val69, Gln73, and Phe108 were noticeable, which may contribute to better ligand–enzyme interaction (Figure 33B).^118^

Regarding γ-secretase inhibitors, studies have described severe side effects associated with the inhibition of Notch processing or accumulation of the carboxy-terminal fragment (β-CTF). In contrast, γ-secretase modulators (GSM’s) reduce pathogenic Aß42 levels without affecting such events. In this context, Sekioka described the synthesis and evaluation of γ-secretase modulatory activity *in vitro* and the pharmacokinetic profile of imidazopyridine derivatives (Figure 34). Most compounds showed significant Aβ42 inhibitory activity (IC_50_ = 0.39 to 9.8 μM), with compound **100** being the best modulator of γ-secretase (IC_50_ = 0.39 μM). The structure–activity relationship (SAR) study showed that the carbonyl group had a significant effect on the GSM activity, where the planar structure formed by the 5-membered ring cyclization (including the 5-membered pseudo-ring) was responsible for the fixation of the direction of the carbonyl, which led to an increase in activity.

**Figure 34:**
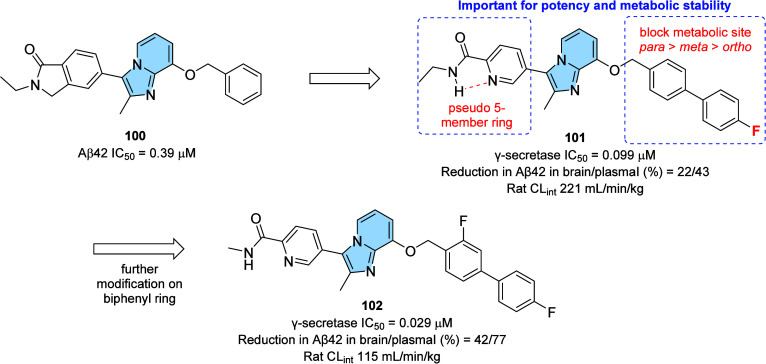
Structural relationships between imidazo­[1,2-*a*]­pyridine derivative (**100**–**102**) modulators of γ-secretase and Aβ42 aggregation.

Furthermore, **100** demonstrated a good pharmacokinetic profile, being metabolically stable (CLint in mice = 611 mL/min/kg), with a good brain/plasma ratio in rats (Kp, brain = 0.72) and did not detectably inhibit the CYP3A4 activity.^119^ Subsequently, the same group^120^ developed the *N*-ethylpyridinyl-2-carboxamide derivative **101**, which showed a superior pharmacokinetic profile and high *in vitro* GSM activity (IC_50_ = 0.099 μM), in addition to high brain exposure and significantly reduced Aβ42 brain levels in mice, without inhibition of CYP3A4 *in vitro*. Subsequent structural optimization of the biphenyl group and the pyridine-2-amide subunit led to the development of derivative **102**, which had the highest GSM activity *in vitro* (IC_50_ = 0.029 μM), undetectable CYP inhibition, and high level of brain exposure. Additionally, **102** demonstrated a reduction in Aβ42 brain levels *in vivo* in rats at an oral dose of 30 mg/kg. Imidazopyridine **102**, at a dose of 10 mg/kg (po) for 8 days, was also able to reverse the cognitive deficit in a rat model of AD. These results suggest that the use of imidazopyridine derivatives such as GSMs is a promising strategy for the treatment of AD.

There are several factors that affect AD etiology. One of the major factors that influences disease progression is neuronal cell inflammation. Damaged neurons and amyloid plaques release several inflammatory cytokines, namely, TNF-α, IL-1β, and COXs. This inflammatory event leads to neuronal cell degeneration and cell death. Hence to combat AD, treatment with anti-inflammatory compounds can serve as a therapeutic weapon. Cyclooxygenase (COX), which plays a role in converting arachidonic acid to inflammatory mediators, could be inhibited by nonsteroidal anti-inflammatory drugs (NSAIDs). In recent years, selective COX-2 inhibitors with a lower incidence of adverse effects attained an important position in medicinal chemistry.^121,122^

Movahed and collaborators^121^ described a new series of 2-(4-(methylsulfonyl)­phenyl)-*N*-phenylimidazo­[1,2-*a*]­pyridin-3-amine as selective COX-2 inhibitors (Figure 35).The designed compounds were synthesized through two-step reactions. with good yields. The compounds were evaluated by an *in vitro* assay against COX-1 and COX-2. All compounds were selective COX-2 inhibitors with selectivity indices (SIs) ranging from 42.3 to 508.6 times and COX-2 IC_50_ values of 0.07–0.39 μM. Compound **103a** showed the highest value of activity (IC_50_ COX-2 = 0.07 μM) and selectivity (SI COX-2 = 508.6). The *in vivo* formalin test was performed to assess the analgesic activity of synthesized compounds. This test indicated that **103b** displayed the highest analgesic activity.

**Figure 35:**
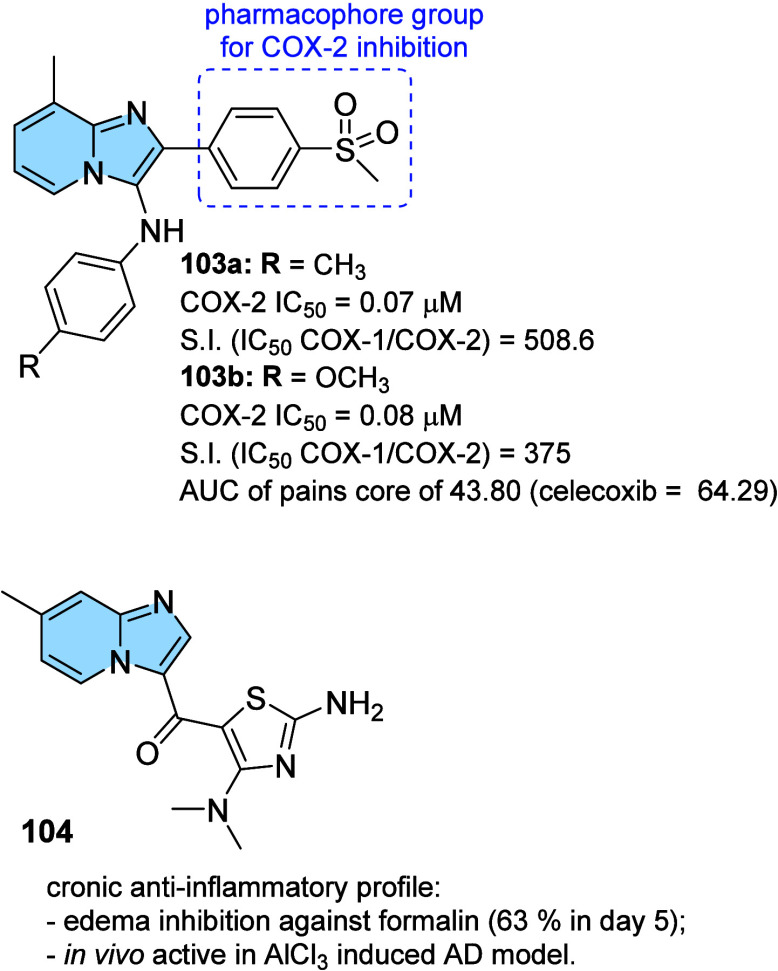
Imidazo­[1,2-*a*]­pyridine derivatives (**103a**, **103b**, and **104**) with anti-inflammatory activity.

Sagar and colleagues^122^ reported a new series of substituted (2-aminothiazol-5-yl)­(imidazo­[1,2- *a*]­pyridin-3-yl)­methanones (Figure 35). These molecules were tested for their anti-inflammatory benefits using *in vivo* acute and chronic inflammation models. Compound **104** exhibited promising anti-inflammatory activity for anti-Alzheimer’s activity in an aluminum chloride (AlCl_3_) model that mimics AD neurotoxicity. Overall, **104** showed promising results by exhibiting antiamnesic, neuroprotective, and antiamyloid activities. Furthermore, compound **104** did not exhibit GI toxicity like other NSAIDS, emerging as a promising lead candidate for the treatment of AD.

### 3.3 Designed against Tuberculosis

Tuberculosis (TB) is an infectious disease usually caused by *Mycobacterium tuberculosis* (MTB). The emergence of drug-resistant MTB strains is a worldwide challenge for the control of this disease, and it is estimated that among new cases, the percentage of resistant strains reaches 19% for standard treatment with rifampicin (RFP). Worldwide, approximately 10.6 million people fell ill with TB in 2021, causing 1.6 million deaths from tuberculosis.^123^ Incorrectly chosen treatments, immunodeficiencies, and social vulnerabilities are some of the parameters associated with the emergence of resistant etiological agents. The progressive increase in recent years of MDR-MTB cases further increases the desire for new chemotherapy alternatives.^124,125^

Recent advances in the development of derivatives containing the imidazo­[1,2-*a*]­pyridine nucleus as potential anti-TB agents and their structure–activity relationship (SAR) led to the identification of the class known as imidazo­[1,2-*a*]­pyridines amides (IPAs), a scaffold that has been showing promising results for MTB strains. As an example, the compound Q203 (**105**), renamed telacebec (Figure 36A), recently completed phase II clinical studies and has QcrB, a subunit of the mycobacterial cytochrome bc1, as its mechanism of action. Telacebec has demonstrated good dose-dependent efficacy in a phase 2A early bactericidal activity (EBA) study in patients with drug-susceptible tuberculosis. The compound has also shown promising activity against Buruli ulcers (Mycobacterium ulcerans), a chronic necrotizing disease of the skin and bone that can cause permanent deformity and long-term disability. Reflecting its therapeutic potential, telacebec has been granted both Orphan Drug Designation and Fast Track Designation by the U.S. FDA.^126^

**Figure 36:**
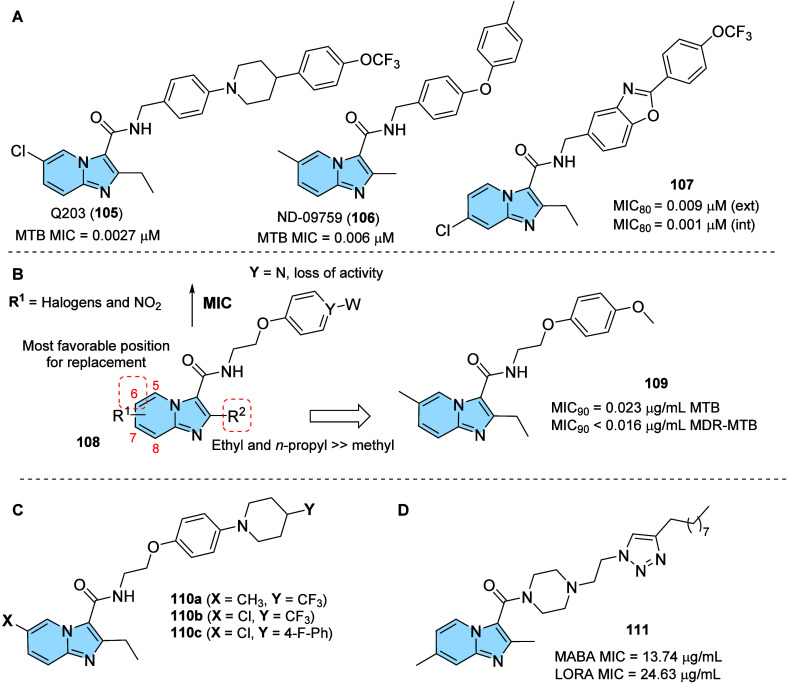
Imidazo­[1,2-*a*]­pyridines amide derivatives (**105**–**109**, **110a–c**, and **111**) with antitubercular activity.

Worth noting, the original article of Q203 (telacebec) (**105**) discovery is the most cited experimental work with imidazo­[1,2-*a*]­pyridines.^127^ Compound **105** and IPA derivative ND-09759 (**106**) (Figure 36A) showed good potency against multidrug-resistant (MDR-MTB)^128–130^ and extensively drug-resistant (XDR-MTB) MTB strains. Derivative IPA **107** (Figure 36A), with a side chain formed by the 1,3-benzodioxole subunit (MIC_80_ = 0.009 μM), also showed excellent pharmacokinetic results for oral administration and a long half-life. Several analogs of IPAs have been synthesized and evaluated to enhance their activities and selectivities as anti-TB agents.^131–138^

Wang presented a study with important structural changes in the known scaffold of IPA derivatives for tuberculosis (**108**) (Figure 36B). Influences in electronic changes were evaluated in the imidazopyridine nuclei, using electron donor and electron-withdrawing substituents in different positions as well as substitutions in the benzene ring of the side chain. For all compounds, the cytotoxic profile (MTB and MDR-MTB) and the pharmacokinetic parameters were monitored. The compounds were initially evaluated *in vitro* in MTB H37Rv strains, and the minimum inhibitory concentrations (MIC_90_) ranged from 0.023 to 32 μg/mL. This parameter evaluates the lowest concentration of the compound responsible for limiting the visible growth of a bacterium to 90%. The most promising analogs were also metabolically evaluated in human hepatocytes, showing relatively high metabolic stability times, *t*_1/2 min_ = 12.9 to 54.2 min. The compounds studied were active for resistant strains, MDR-MTB 11168 and 9160, clinically isolated (MIC_90_ < 0.016 at 1 μg/mL), with low oral acute lethal toxicity and more potent than the drugs used in the clinic, isoniazid (INH), MIC_90_ > 40 μg/mL, and rifampicin (RFP), MIC_90_ = 36.889 μg/mL. Among the compounds that stood out, the derivative IPA **109** was identified as one of the most promising (Figure 36B).

Li and co-workers described studies of structure–activity relationships in IPA derivatives in which the presence of a more flexible side chain, the presence of a cyclic amine, and the different isosteres of piperidine were evaluated. The studies led to promising compounds (MIC < 0.016 μg/mL), with activities similar to Q203 (**105**) and better than two drugs used in MTB treatments, such as INH, MIC = 0.049 μg/mL, and RFP, MIC = 0.050 μg/mL. Additionally, studies were performed on multidrug-resistant strains, clinical isolates, resistant to INH and RFP. The compounds that showed activity for MTB strains also showed strong activity in the MDR-MTB strains used (MIC = 0.002 to 0.030 μg/mL), with high selectivity, evaluated in Vero cells (CC_50_ > 64 μg/mL). Analog IPAs **110a**–**c** (MIC < 0.002 μg/mL) (Figure 36C) were identified as the most promising antitubercular candidates, with high potency and selectivity, in addition to having the best pharmacokinetic profiles in mice.

Still based on the IPA scaffold, Nandikolla and colleagues proposed the incorporation of a piperazinyl-1,2,3-triazole pharmacophore.^139^ The compounds obtained were then evaluated *in vitro* against the MTB H37Rv strain in replication (MABA method) and nonreplicating (LORA method) to determine their antimycobacterial activity. The antitubercular activities obtained by the compounds were quite distinct from each other, with compound **111** (Figure 36D) being the most active for both methods (MABA: MIC = 13.74 μg/mL and LORA: MIC = 24.63 μg/mL). A SAR study carried out among the obtained derivatives showed that the substitution of the aliphatic group in the triazole nucleus favors the antitubercular activity when compared to compounds with aromatic substitution. The molecular modeling study of compound **111** allowed the observation of a hydrogen-bonding interaction between imidazopyridine nitrogen and amino acid residue GLY-100, which corroborates the antimycobacterial activity observed for this compound. Furthermore, the ADMET parameters of compound **111** were evaluated *in silico*, where the results were promising.

Treatment for MDR-TB and XDR-TB has so far proven to be lengthy, expensive, and difficult to manage. According to reports, treatment success in XDR-TB does not reach 20%, with death and failure percentages reaching 49% of patients. A new mechanism of action related to the energy profile of mycobacteria, with ATP synthase inhibition, has been explored for anti-TB compounds after FDA approval of bedaquiline, a quinoline derivative that showed activities against MDR-TB and XDR-TB.^140^ Tantry and colleagues identified new anti-TB through HTS performed with 900,000 compounds, in which possible inhibitors of oxidative phosphorylation (ATP synthesis) were identified.^138^ The study identified imidazo­[1,2-*a*]­pyridine ethers (IPEs) (Figure 37) with high potency in inhibiting ATP synthesis (IC_50_ = 0.005 to 36.1 μM) and their correlation with anti-TB activity (MIC = 0.03 to >250 μM). The SAR study for the IPEs derivatives defined that the substitution in the imidazopyridine nucleus with electronegative and hydrophobic character, leading to compound **112a**, was favorable to the activity, as well as the presence of methyl in compound **112b**, which was considered equipotent, with a gain of up to 8-fold in inhibition of ATP synthesis and 3-fold in MTB activity relative to the unsubstituted compound (ATPS IC_50_ = 0.13 μM; MIC = 1.6 μM). Additionally, compound **112c** showed good antituberculosis activity (MIC = 0.03 μM), with excellent inhibition of ATP synthesis (IC_50_ = 0.005 μM) and excellent selectivity with a toxicity index above 1000.

**Figure 37:**
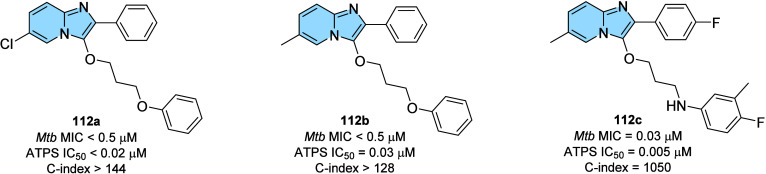
Imidazo­[1,2-*a*]­pyridine ether derivatives (**112a**–**c**) with promising antitubercular activity.

Zolpidem (**8**) is a drug used to treat insomnia and which also has antitubercular activity, so its structure has been used as a framework for studies to potentiate this activity (*Mtb* MIC = 10 μM) and improve its metabolic profile and pharmacokinetics.^141,142^ In this context, Reddyrajula and colleagues described structural modifications in Zolpidem (**8**) exploiting the presence of the 1,2,3-triazole nucleus, a pharmacophore described for anti-TB activity.^143–146^ Derivatives **113a** (*Mtb* MIC = 4.36 μM) and **113b** (*Mtb* MIC = 4.33 μM) (Figure 38) stood out as the most active compounds. Molecular docking studies, with a target already validated for anti-TB, InhA of MTB, revealed important interactions of these ligands with the target enzyme, such as hydrogen bonds, hydrophobic π-stacking interactions, and interaction with residue Tyr158. These derivatives also showed high selectivity with low toxicity in Vero cells with IS ranging from 76.7 (**113b**) to 378.2 (**113a**) (Figure 38).

**Figure 38:**
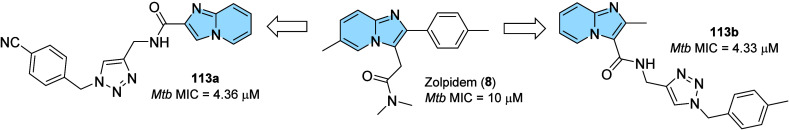
Zolpidem-based derivatives with anti-TB activity.

### 3.4 Designed against Neglected Tropical Diseases

Neglected tropical diseases (NTDs) are a set of diseases intrinsically related to poverty with occurrences described in 149 countries and that affect more than 1.4 billion people, 500 million of which are children.^147–149^ According to the WHO, they are a heterogeneous group consisting of 20 diseases caused by parasites, viruses, and bacteria. They present as the main vectors for its incidence and the difficulties in the access to basic sanitation, drinking water, and health.^147,148^ NTDs are often described as chronic, debilitating diseases with the ability to promote poverty because of their effects on women’s productivity, child development, social stigma, and health. Even representing 11% of the global disease burden, only 1% of new therapeutically approved chemical entities between 2000 and 2011 were directed to neglected diseases.^149,150^

According to the WHO, leishmaniasis is among the main neglected diseases in the world. Characterized by diversity and complexity, more than 20 species of Leishmania are transmitted to humans by about 30 species of mosquitoes.^150–152^ The constant research and development of new antileishmanial compounds are essential, mainly due to problems related to drug resistance, toxicity, phenotypic variety of species, and limited treatment options.^153,154^

The nitro group is present in the structure of several compounds under development for the treatment of leishmaniasis, such as delamanid, an antitubercular drug that has shown promising results for the treatment of leishmania infections.^155–157^ The nitro group is bioactivated selectively in the parasite, carried out by enzymes known as nitro-reductases (NTRs).^158^ The 3-nitro-imidazo­[1,2-*a*]­pyridine nucleus has been studied and explored for the development of antiparasitic drugs, with interesting initial results of potency and selectivity.^153,154,159–161^ In this context, the works from Fersing and collaborators^153,161^ showed promising results for the antileishmanial activity of 3-nitro-imidazo­[1,2-*a*]­pyridine derivatives inspired by fexinidazole (**114**) (Figure 39).

**Figure 39:**
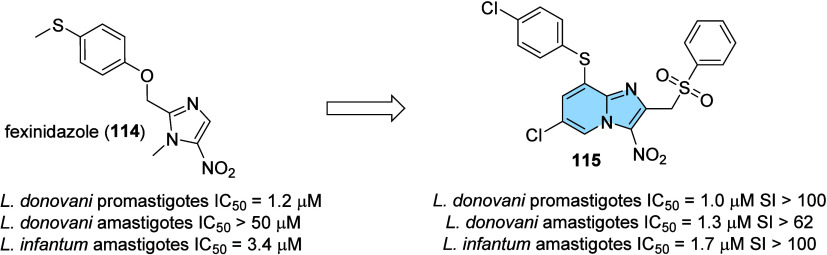
3-Nitro-imidazo­[1,2-*a*]­pyridine derivative (**115**) with antileishmanial activity designed from fexinidazole (**114**).

Among the most promising derivatives, **115** was active and selective against *L. donovani* promastigotes (IC_50_ = 1.0 μM; SI > 100), *L. infantum* amastigotes IC_50_ = 1.7 μM (SI > 100), and *L. donovani* amastigotes IC_50_ = 1.3 μM (SI > 62.5). Additionally, all of the derivatives studied showed possible metabolism in type 1 nitroreductase (NTR1), a mechanism similar to that of the precursor fexinidazole (**114**) (Figure 39). *In vivo* studies have also shown that compound **115** is not mutagenic or genotoxic, further demonstrating a reduced microsomal half-life.

Nandikolla et al., based on some leishmanicidal compounds described in the literature (**116–120**)^160–163^ and using molecular hybridization as Medicinal Chemistry strategy, designed new imidazo­[1,2-*a*]­pyridine derivatives (**121–122**) as possible agents for the treatment of leishmaniasis^152^ (Figure 40). Thirty-five analogs were synthesized and evaluated, and among them, five (**121a**–**b**; **122a**–**c**) showed moderate activity against *L. major* promastigotes, with IC_50_ ranging from 15 to 47 μM and low selectivity (SI = 1.57 to 6.37). This work presented a real limitation of molecular hybridization as a tool for designing new drugs. Although structural design has been based on compounds with excellent antileishmanial activities,^164–166^ the evaluated derivatives with imidazo­[1,2-*a*]­pyridine scaffold were not able to reproduce the same potential of their precursors, at least against the tested strain.

**Figure 40:**
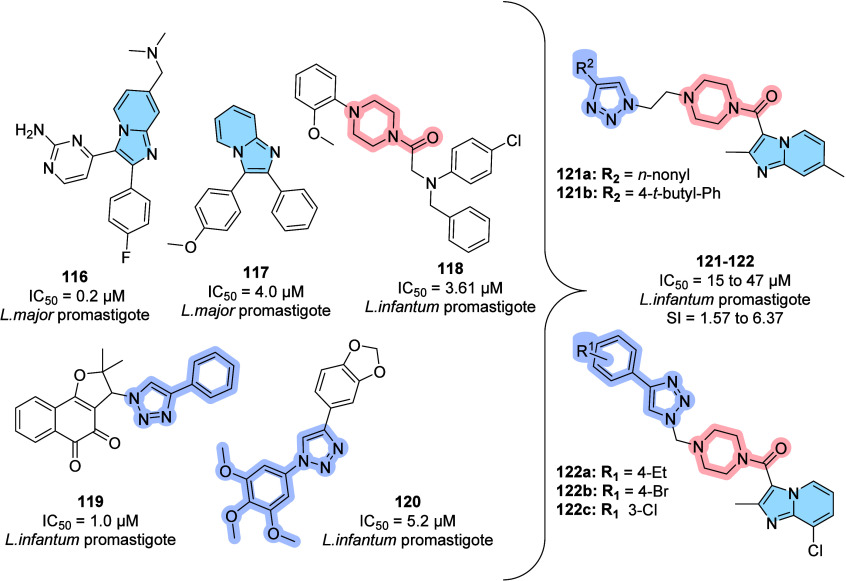
Hybrid derivatives of imidazo­[1,2-*a*]­pyridines (**121a**–**b**; **122a**–**c**) with antileishmanial activity.

The neglected diseases caused by trypanosomatid parasites are of great relevance among NTDs; they are the above-described leishmaniasis,^152^ and human African trypanosomiasis (HAT), endemic in 36 countries and a consequence of infection by *Trypanosoma brucei* (*T. brucei*)^148,156,167^ and Chagas disease (CD), related to an infection caused by *Trypanosoma cruzi* (*T. cruzi*).^159^

Pharmacological treatment for CD is restricted to two nitroheterocyclic drugs introduced in the 60s–80s, benznidazole (Bz) and nifurtimox (Nif). The results obtained with Nif and Bz vary according to the stage of the disease, the period and dose of treatment, the age, and the geographic origin. Significant results in the acute phase of the disease are reported, but its effectiveness declines with the advancement of infection. The high rate of side effects, especially in adults, has led to treatment abandonment in many situations, while children have a greater tolerance to the medication.^159,164,168^

Silva and collaborators^167^ described new bioisosteres of the benzothiazole derivative DAP (**123**) (IC_50_ = 35 nM, *T. brucei*)^169^ (Figure 41) with EC_50_ against *T. cruzi* of up to 0.09 μM and against *T. brucei* of up to 0.02 μM. The difluorinated derivative (**124**) had a potent EC_50_ against both strains, with EC_50_ of 0.39 and 0.16 μM against *T. cruzi* and *T. brucei*, respectively. The SAR showed that the insertion of the pyrimidine nucleus resulted in the most active candidate (**125**), with an EC_50_ of 0.09 μM for *T. cruzi* and an EC_50_ of 0.02 μM for *T. brucei*. Analog **125** also exhibited low toxicity for the CRL-8155 cell line with an EC_50_ greater than 50 μM. Compound **125** further demonstrated parasite *in vivo* inhibition comparable to the DAP (**123**) treatment group. Results of metabolic stability and pharmacokinetic properties further corroborated the auspiciousness of this compound as an antitrypanosomal active ingredient.

**Figure 41:**
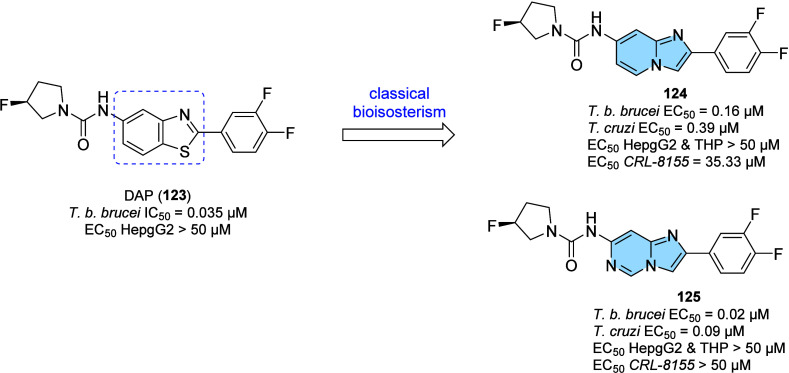
Imidazo­[1,2-*a*]­pyridine derivatives with antitrypanosomal activity designed from DAP (**123**).

In November 2018, the European Agency’s Committee on Medicines for Human Use (CHMP) authorized the use of fexinidazole (**114**), in oral form, for the treatment of Gambian human African trypanosomiasis (gHAT).^156,170^ Since then, numerous imidazole correlate derivatives have been tested and evaluated for trypanosomiasis parasitic diseases.^152,159,167^ The biological activity of nitroheterocyclic compounds involves the formation of electrophilic metabolites that react with cellular components, such as DNA or proteins, to form cytotoxic covalent adducts.^158,171^ Fersing and colleagues^159^ described the design of antitrypanosomal 3-nitro-imidazo­[1,2-*a*]­pyridine derivatives (Figure 42). Pharmacomodulation at position 8 of the imidazo­[1,2-*a*]­pyridine scaffold, using the Sonogashira cross-coupling reaction, resulted in 11 novel derivatives with remarkable *T. brucei* activities (0.04 ≤ EC_50_ ≤ 0.16 μM) and, in general, high-selectivity indices (9 ≤ SI ≤ 1786). The nitro compound (**127**), EC_50_ = 0.07 μM, was much more active than its corresponding amino derivative (**126**), EC_50_ = 6.8 μM, indicating the fundamental role of the presence of the nitro group in the pharmacophore. The mechanism of action involves the metabolism of type 1 nitro-reductases. *In vitro* physicochemical studies and pharmacokinetic parameters for molecule **127** showed interesting properties when compared to other 3-nitroimidazo­[1,2-*a*]­pyridine published derivatives, mainly regarding water solubility and microsomal stability. *In vivo* tests showed a plasma half-life of 10 h, with good results for oral administration in mice. Compound **127** still showed moderate activity against *L. donovani* promastigote, EC_50_ = 7.4 μM, and in *T. cruzi* amastigotes, EC_50_ = 1.2 μM, standing out as an important imidazo­[1,2-*a*]­pyridine derivative with antitrypanosomal activity.

**Figure 42:**
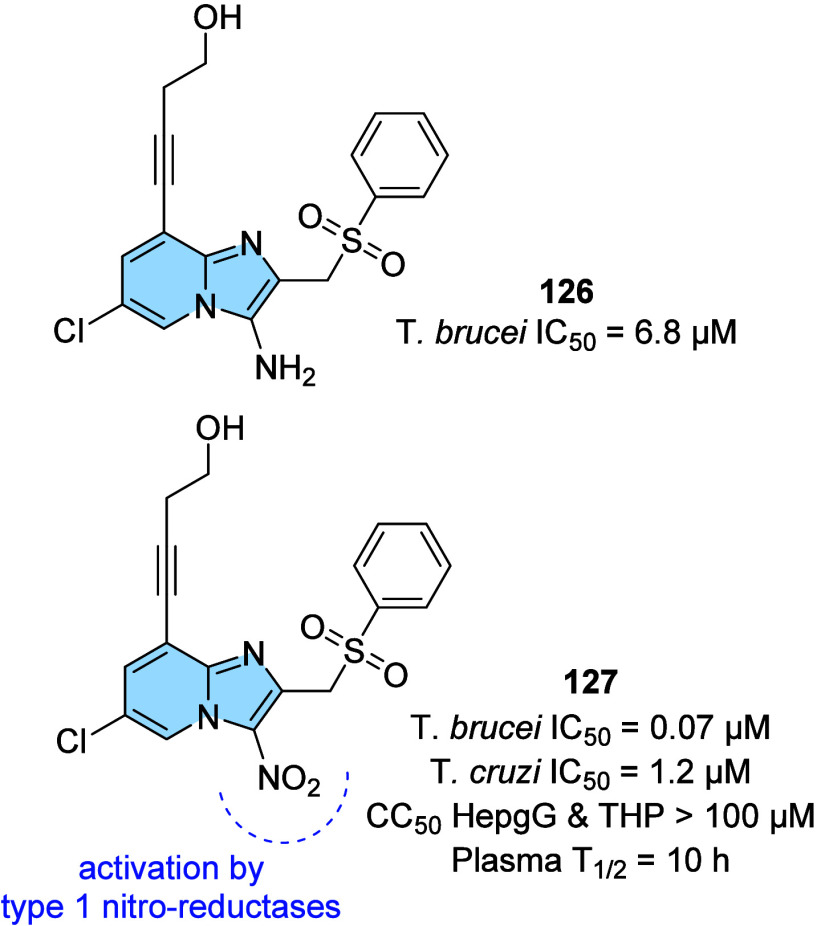
8-Alkynyl-3-nitro-imidazo­[1,2-*a*]­pyridine derivatives with antitrypanosomal activity.

Malaria is an infectious disease transmitted by mosquitoes and is caused by parasitic protozoa of the genus Plasmodium. Five species are known to cause the disease: *P. falciparum*, *P. vivax*, *P. ovale*, *P. malariae*, and *P. knowlesi*. The first two are the most incident, with characteristics different from those of the host. *P. falciparum* is considered the most lethal form, with high death rates; the others are classified as nonlethal, but present irreversible damage to the hosts.^172^

Studies with the imidazo­[1,2-*a*]­pyridine nucleus have generated important reports on antimalarial activities.^173–176^ Among the biological targets studied in the disease life cycle, *PfPKG*-type-dependent protein kinases are highlighted as an essential and selective target for the parasite.^173,174^ Large and colleagues^177^ studied imidazo­[1,2-*a*]­pyridine derivatives (**128**) (Figure 43) as possible *PfPKG* kinase inhibitors for the treatment of malaria. At first, substitutions were compared based on two biological parameters: kinase inhibition (*PfPKG* pCI_50_ between 6.07 to 8.70) and ligand lipophilicity efficiency (LLE between 4.2 to 6.3). Pyrimidine derivative **128d** (*PfPKG* pIC_50_ = 7.92; LLE = 5.9) showed inhibition results comparable to those of derivative **128a** (*PfPKG* pIC_50_ = 8.70; LLE = 6.2), which were the best derivatives of series I. Molecular docking studies carried out on the crystal structure *PfPKG* (PDB: 5DYK) with series I compounds pointed to the classical interactions of guanidines with the kinase residue Val621, as well as more favorable angles (smaller out-of-plane rotation of the bicyclic nucleus) of the **128a** derivative with the target, explaining its better activity. Aiming at a possible metabolic improvement, the series II compounds were designed by removing the tertiary amine from the imidazo­[1,2-*a*]­pyridine nucleus. Compounds **128b** (*PfPKG* pIC_50_ = 8.60; LLE = 4.60; Pf HXI pEC_50_ = 6.94) and **128c** (*PfPKG* pIC_50_ = 8.30; LLE = 5.00; pEC_50_ = 6.80) showed the best series II biochemical and antimalarial profiles. The studied derivatives **128a** and **128b** were considered antimalarial hits by the author.

**Figure 43:**
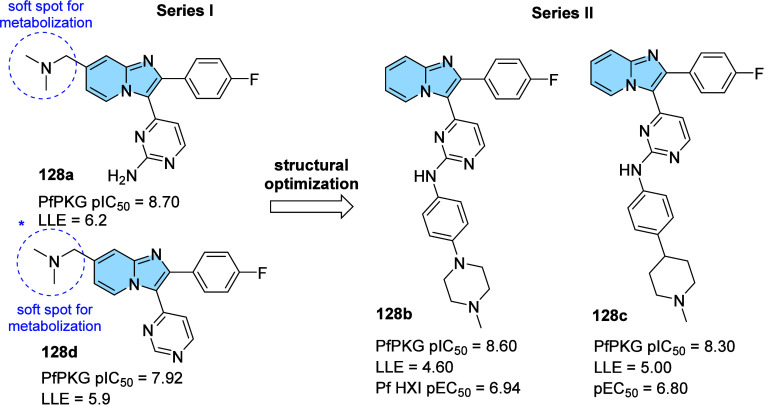
Structural optimization of imidazo­[1,2-*a*]­pyridine derivatives with antimalarial activity.

The Zika virus (ZIKV), a mosquito-borne flavivirus primarily transmitted by *Aedes aegypti* and *Aedes albopictus*, garnered global attention following outbreaks in the Americas during 2015–2016.^178^ A recent epidemic highlighted its potential for severe neurological complications, including congenital Zika syndrome and Guillain-Barré syndrome.^178^ Advances in understanding the molecular mechanisms of ZIKV pathogenicity and immune response are crucial for treatment development and vector control strategies.^179^ As ZIKV continues to pose a threat, especially with climate change influencing vector distribution, ongoing surveillance and research are essential for mitigating future outbreaks.

Arruda and collaborators described the synthesis and characterization of a series of coumarin-imidazo­[1,2-*a*]­pyridine hybrids and their corresponding complexes with Zn­(II).^180^ These molecules were evaluated for their antiviral activity against ZIKV. The compounds exhibited potent antiviral activity, with EC_50_ values ranging from 0.55 to 4.8 μM and a selectivity index (SI) of up to 1490. Notably, the complexes, which were the first anti-ZIKV Zn­(II) complexes of this class, generally showed enhanced activity compared to their respective ligands, with some compounds outperforming the reference antiviral, ribavirin. Time-of-addition assays suggested that some compounds interfere with both the entry (with a virucidal component) and viral replication phases, while others act only on the replication phases. Ligand **129** and complex **130** stood out by demonstrating virucidal activity, which makes them promising candidates as potential preventive compounds for ZIKV infection (Figure 44).^180^ Compound **129** was the most potent (EC_50_ = 0.55 μM) and most selective (SI = 1490) compound. Among the complexes, **130** showed the highest potency (EC_50_ = 0.77 μM) and SI (828). Molecular modeling (docking) studies suggested that the molecules may have dual modes of action. They indicate possible interactions with the ZIKV envelope protein (important for viral entry) and with the ZIKV NS2B-NS3 protease complex (essential for replication).

**Figure 44:**
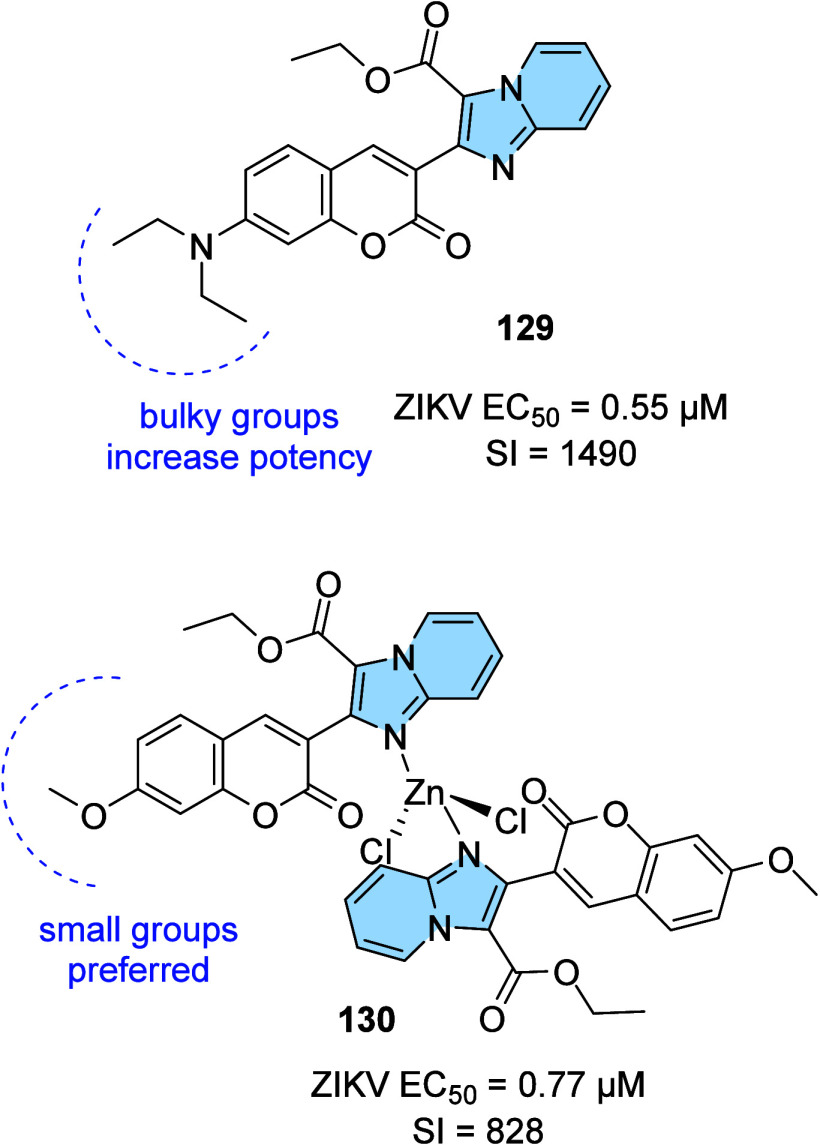
Coumarin-imidazo­[1,2-*a*]­pyridine derivatives with anti-ZIKV activity.

## 4 Conclusions

Based on the extensive occurrence of studies related to the synthesis and pharmacological evaluation of imidazo­[1,2-*a*]­pyridine (IM) analogues in recent literature, this review highlighted the continued relevance of IMs as a versatile and promising heterocyclic scaffold in Medicinal Chemistry. The ease of synthesis and the diversity of available methods, from classical condensation reactions and multicomponent reactions (such as GBB) to more recent approaches involving couplings and intramolecular cyclizations, enabled access to a wide range of IM derivatives. This structural diversity allows for the modulation of physicochemical and pharmacological properties, culminating in the identification of multiple derivatives with high potency and selectivity against specific biological targets.

An analysis of scientific output between 2017 and 2024 demonstrated a growing and focused interest in the therapeutic potential of IMs, with a predominant focus on four main areas: cancer (38%), central nervous system disorders (20%), microbiology (11%), and parasitology (7%).

Table 1 summarizes the main bioactive molecules containing the imidazo­[1,2-*a*]­pyridine scaffold, their biological targets, and reported activities from 2017 to 2025. In oncology, IMs have emerged as inhibitors of crucial targets, such as tubulin, HDACs (especially HDAC6), various kinases (PI3Kα, c-Met, Nek2, Aurora B, FLT3), transcriptional regulators (STAT3, ENL), and immune checkpoints (PD-1/PD-L1). In research on neurodegenerative diseases such as Alzheimer’s, derivatives have been developed with multitarget activities, including the inhibition of cholinesterases (AChE/BuChE), secretases (BACE1), and modulation of γ-secretase, in addition to showing anti-inflammatory potential by inhibiting COX-2.

**Table 1:**
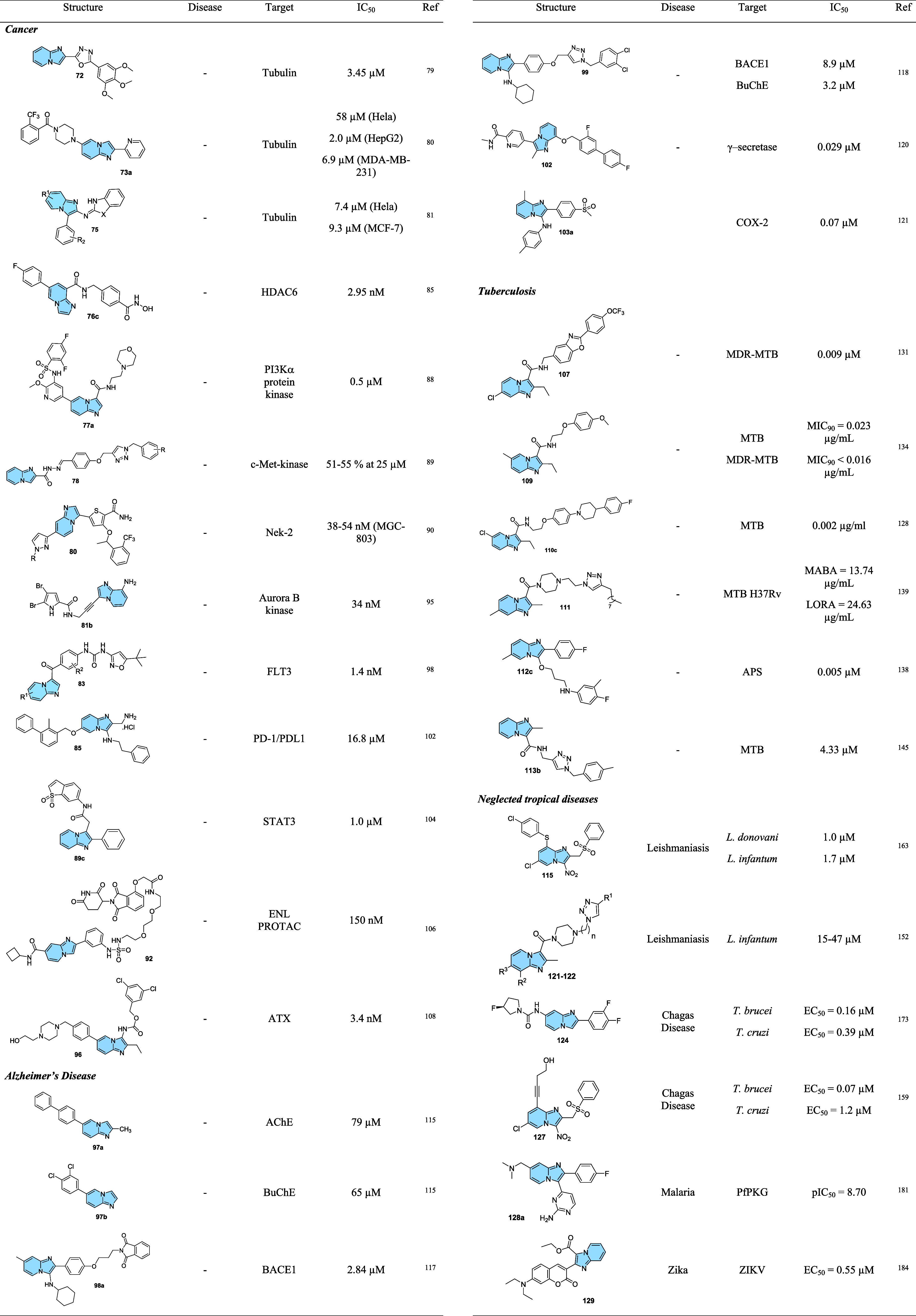
Summary of the Main Bioactive Molecules Containing the Imidazo­[1,2-*a*]­pyridine Scaffold, Their Biological Targets, and Their Reported Activities from 2017 to 2025

In the field of infectious diseases, IMs continue to be a key scaffold, particularly in the development of antituberculosis agents. Derivatives such as imidazo­[1,2-*a*]­pyridine amides (IPAs), including the clinical candidate Q203 (**105**), and imidazo­[1,2-*a*]­pyridine ethers (IPEs) have shown remarkable potency against multidrug-resistant (MDR-MTB) and extensively drug-resistant (XDR-MTB) strains of *Mycobacterium tuberculosis*, acting on targets such as the cytochrome bc1 complex and ATP synthesis. Furthermore, the 3-nitro-imidazo­[1,2-*a*]­pyridine core has proven particularly promising against neglected tropical diseases, with derivatives exhibiting potent activity against Leishmania and Trypanosoma. More recently, imidazo­[1,2-*a*]­pyridine hybrids have also been identified as potent dual inhibitors (entry and replication) of Zika virus.

Despite that imidazo­[1,2-*a*]­pyridine has been considered a privileged structure,^181^ and the existence of several marketed drugs containing the imidazo­[1,2-*a*]­pyridine core, this scaffold has not been present in any recently launched drug.^182^

As future directions and given this promising biological profile, the integration of computational drug design and AI-assisted screening could offer a compelling route to accelerate, focus, and derisk drug-discovery efforts based on the imidazo­[1,2-*a*]­pyridine core. Indeed, a recent collaborative virtual screening campaign exploited five proprietary pharmaceutical libraries to expand an imidazo­[1,2-*a*]­pyridine hit series for visceral leishmaniasis: the *in silico* examination named “Booster process” significantly improved the selectivity index and antiparasitic potency of the series.^151^

Despite the imidazo­[1,2-*a*]­pyridine nucleus having been widely incorporated into many marketed drugs, indicating the safety of this scaffold, challenges in PK and toxicity always remain central obstacles. Incomplete understanding of ADME of new decorated imidazo­[1,2-*a*]­pyridine can often limit accurate prediction of human exposure and safety. The new decorations can arise from toxic effects from new off-target interactions, immune responses, or reactive metabolite formation. Although advances such as physiologically based pharmacokinetic (PBPK) modeling and early toxicity biomarkers improve prediction, significant gaps persist, particularly for emerging new compounds and targets. In this scenario, the AI-assisted new methodologies described above will be very helpful in the future.

As a lesson, future research should focus on deepening the understanding of mechanisms of action, exploring new structural combinations and biological targets, and employing multidisciplinary approaches, such as the integration of computational modeling, high-throughput screening, and detailed pharmacokinetic studies, to accelerate the discovery and development of new IM-based drug candidates. The remarkable synthetic flexibility and the broad spectrum of biological activity firmly establish imidazo­[1,2-*a*]­pyridine as a privileged scaffold whose therapeutic potential is far from being exhausted.

## 1

Chemistry, Universidade Federal Fluminense, Saint John the Baptist Hill Street, Niterói, Rio de Janeiro, 24220–900, Brazil
